# Formation and current-induced motion of synthetic antiferromagnetic skyrmion bubbles

**DOI:** 10.1038/s41467-019-13182-6

**Published:** 2019-11-14

**Authors:** Takaaki Dohi, Samik DuttaGupta, Shunsuke Fukami, Hideo Ohno

**Affiliations:** 1 Laboratory for Nanoelectronics and Spintronics, Research Institute of Electrical Communication, Tohoku University, 2-1-1 Katahira, Aoba, Sendai, 980-8577 Japan; 2 Center for Science and Innovation in Spintronics, Tohoku University, 2-1-1 Katahira, Aoba, Sendai, 980-8577 Japan; 3 Center for Spintronics Research Network, Tohoku University, 2-1-1 Katahira, Aoba, Sendai, 980-8577 Japan; 4 Center for Innovative Integrated Electronic Systems, Tohoku University, 468-1 Aramaki Aza Aoba, Sendai, 980-0845 Japan; 5 WPI Advanced Institute for Materials Research, Tohoku University, 2-1-1 Katahira, Aoba, Sendai, 980-8577 Japan

**Keywords:** Electrical and electronic engineering, Spintronics, Spintronics

## Abstract

Skyrmion, a topologically-protected soliton, is known to emerge via electron spin in various magnetic materials. The magnetic skyrmion can be driven by low current density and has a potential to be stabilized in nanoscale, offering new directions of spintronics. However, there remain some fundamental issues in widely-studied ferromagnetic systems, which include a difficulty to realize stable ultrasmall skyrmions at room temperature, presence of the skyrmion Hall effect, and limitation of velocity owing to the topological charge. Here we show skyrmion bubbles in a synthetic antiferromagnetic coupled multilayer that are free from the above issues. Additive Dzyaloshinskii-Moriya interaction and spin-orbit torque (SOT) of the tailored stack allow stable skyrmion bubbles at room temperature, significantly smaller threshold current density or higher speed for motion, and negligible skyrmion Hall effect, with a potential to be scaled down to nanometer dimensions. The results offer a promising pathway toward nanoscale and energy-efficient skyrmion-based devices.

## Abstract (web summary)

Realizing stable, room-temperature magnetic skyrmions that can be moved along current is challenging. Here, the authors observe skyrmion bubbles in a synthetic antiferromagnetic coupled multilayer at room temperature and demonstrate its current-induced motion free from the skyrmion Hall effect.

## Introduction

Skyrmion, a quasi-particle introduced by Skyrme^1^, represents topologically protected soliton and is known to emerge in various fields including nuclear physics, optics, liquid crystals, and Bose–Einstein condensation systems. Magnetic skyrmion, stabilized via electron spin^2^, was firstly demonstrated in B20 compounds^3,4^, and then observed in various systems^5,6^ at low temperature. It has been revealed that the magnetic skyrmions exhibit a variety of exotic spin-dependent phenomena through an emergent field generated by its topology in real space^7,8^. Room-temperature magnetic skyrmion has been also observed in heavy metal/ferromagnet heterostructures with broken inversion symmetry, owing to an interfacial Dzyaloshinskii-Moriya interaction (DMI) induced by strong spin-orbit interaction^9–12^. The spin-orbit interaction also gives rise to spin-orbit torque (SOT) under in-plane current application, allowing efficient control of the skyrmion^11^, which shows promise for future spintronic devices^13–15^. However, extensive efforts to realize the skyrmion-based devices such as race-track memory^8,13^ have delineated fundamental issues. For instance, skyrmion Hall effect (SkHE), a diagonal motion of skyrmion with respect to the current direction via the Magnus force^16^ owing to the finite topological charge, is an obstacle when one utilizes skyrmions as information carriers moving on a track^13^. Theoretical studies predicted a SkHE-free motion for antiferromagnetic skyrmions because of the topological charge with opposite sign at each sublattice^16,17^. Skyrmion in ferrimagnetic alloys have been recently reported^18–20^ and current-induced motion without SkHE is demonstrated at a specific temperature where the angular-momentum is compensated, but not in a fully compensated antiferromagnetic system.

Here we show SkHE-free motion of skyrmion bubbles at room temperature in synthetic antiferromagnetic (SyAF) systems, which can be driven with much smaller current density than ferromagnetic skyrmion bubbles. The achieved favorable properties are attributed to the employed stack structure which is engineered so that the Ruderman-Kittel-Kasuya-Yosida (RKKY) interaction^21^, DMI, and SOT act in a concerted way efficiently.

## Results

### Characterization of magnetic properties and domain patterns

**Fig. 1:**
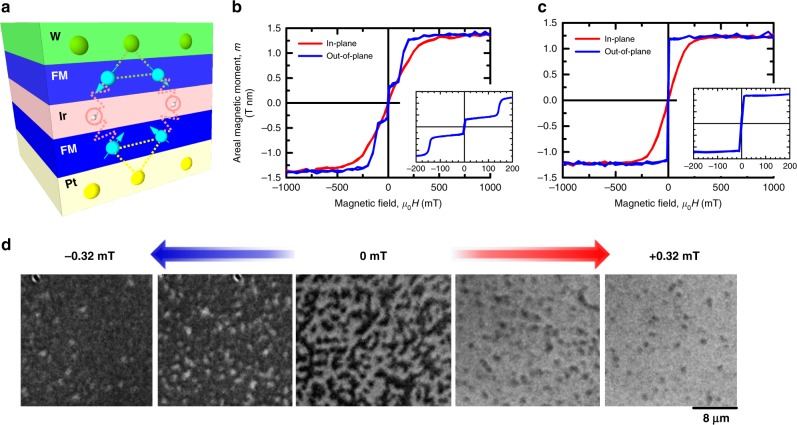
Stack structure, magnetic properties and domain patterns. **a** Schematic of stack structure with synthetic antiferromagnetic coupling and Dzyaloshinskii-Moriya interaction. **b** *m*-*H* curves for stack 1 and **c** stack 2. Blue and red colors denote the curves along out-of-plane and in-plane directions, respectively. **d** Magnetic domain patterns observed by magneto-optical polar Kerr effect microscopy under magnetic fields of −0.32, −0.2, 0, +0.2 mT, and +0.32 mT for stack 1

Figure 1a shows a schematic of the stack structure with SyAF coupling. The stack consists of Ta(1.0)/Pt(4.0)/Co(0.2)/Co_0.19_Fe_0.56_B_0.25_(0.925)/Ir(1.3)/Co(0.375)/Co_0.19_Fe_0.56_B_0.25_(0.6)/W(4.0)/Ru(1.0) (in nm) (stack 1 hereafter). Ir thickness is optimized to obtain maximum SyAF coupling (see Supplementary Note 1). As a reference, a conventional single ferromagnetic stack consisting of Ta(1.0)/Pt(4.0)/Co(0.375)/Co_0.19_Fe_0.56_B_0.25_(0.9)/Ir(1.0)/Ru(1.0) (stack 2 hereafter) is also prepared (see Methods for more details). Magnetization hysteresis curves (*m*–*H* curves) for each stack are shown in Fig. 1b, c. Both stacks have a perpendicular easy axis. As for the stack 2, out-of-plane *m*-*H* curve shows almost no remanence, indicating a formation of multi-domain state at zero fields. For the stack 1, areal magnetic moment decreases abruptly down to 30% from the saturation state at around the perpendicular field *μ*_0_*H*_*z*_ = ±150 mT (*μ*_0_ is the permeability in vacuum), indicating a SyAF coupling between the two ferromagnetic parts through the Ir layer via the RKKY interaction. The coupling energy −*J*_int_ = −*H*_int_(*m*_S_ − *m*_Com_) is obtained to be −0.13 mJ m^−2^, where *H*_int_, *m*_S_, and *m*_Com_ denote perpendicular magnetic field at compensated point, magnetic moments at saturation and at compensated states, respectively. We note that although a fully compensated structure can be prepared (see Supplementary Note 1), we intentionally design the stack to have a small uncompensated moment so that magnetic patterns can be visualized using a magneto-optical Kerr effect (MOKE) microscope. The effect of the degree of compensation on the nature of skyrmions as well as their dynamics will be discussed later in detail. Importantly, we find that the stack 1 also shows no remanence as in the stack 2, implying a formation of multi-domain state with SyAF coupling. Figure 1d shows the magnetic domain patterns under various magnetic fields observed with MOKE microscope. Multi-domain state is observed at zero fields as expected, which transforms into skyrmion-like bubble state with increasing the magnitude of *H*_*z*_, and then to a uniform state. In general, multi-domain state is not easily formed in SyAF systems owing to minuscule dipole interaction. In the present stack, on the other hand, large DMI induced at Pt and W interfaces with the same (left-handed/counter-clockwise) chirality decreases the formation energy of domain walls (DWs), leading to stable multi-domain states at room temperature (see Supplementary Note 2 and Note 3 for quantification of DMI of the each part of the stack). Observation under various perpendicular fields reveals that the size of these skyrmion-like bubbles is insensitive to the fields in the range where such a bubble structure is formed (see Supplementary Note 4). The obtained skyrmion-like bubble is expected to be moved by current efficiently with much-reduced SkHE due to the effectively compensated topological charge.

### Current-induced motion of SyAF skyrmion bubbles

**Fig. 2:**
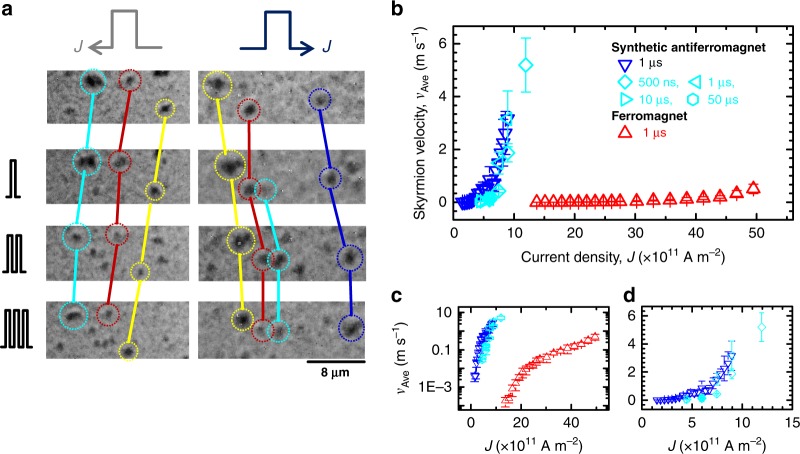
Current-induced motion of synthetic antiferromagnetic skyrmion bubbles. **a** Magnetic state of synthetic antiferromagnetically coupled wire obtained with magneto-optical polar Kerr effect microscopy under current pulse applications at *μ*_0_*H*_*z*_ = 0.2 mT. Each skyrmion is featured by different colors. Current density and pulse width are 7.1 × 10^11^ A m^−2^ and 1 μs, respectively. **b** Current density dependence of average velocity of skyrmion bubbles in stacks 1 (synthetic antiferromagnet) and 2 (ferromagnet), represented by blue and red, respectively. For stack 1, results with various pulse widths are plotted by different symbols. **c** The same result shown by a logarithm scale for vertical axis. **d** The same result focused in a low-current-density region

To confirm whether the observed skyrmion-like bubbles are indeed topologically protected and driven by current efficiently, we next examine the response of these magnetic objects to current. Figure 2a shows a series of MOKE image under current applications. We find that the skyrmion-like bubbles are moved by current pulses in the same direction to the current flow, evidencing that they are topologically protected with chiral Néel profile as expected and their displacement is governed by SOT^11^. Because our stack has negative DMI forming a left-handed/counter-clockwise chiral Néel DWs, the motion along the current direction indicates a positive effective spin Hall angle if the spin Hall effect dominates the SOT. The formation of SyAF coupled and topologically non-trivial skyrmions with the same length scale is reproduced in a micromagnetic simulation with the experimentally determined DMI constant and interlayer exchange coupling (see Supplementary Note 4 for the results of micromagnetic simulation). We also note that there are some bubbles which are nucleated or annihilated under current application, presumably due to thermal and/or pinning effects as has been observed in ferromagnetic systems^11^. Figure 2b shows current density *J* dependence of average velocity *v*_Ave_ for the skyrmion bubbles in the stacks 1 and 2 (see Methods for details). We find that the skyrmion bubbles in SyAF system are driven by one-order-of-magnitude smaller *J* than those in the ferromagnetic system at comparable velocity. We here note that the detectable speed of the skyrmion bubbles is limited by technical reasons, e.g., shortest pulse width (500 ns) and maximum size of the field of view of our setup; the bubbles appear to move with much faster speed than the results presented here when we apply larger current-density pulse even though they are not traceable. We also confirm that traveling distance of skyrmions is proportional to the pulse width (see Supplementary Note 5) and velocities obtained by different pulse widths collapse onto a single trend (light blue plots in Fig. 2b), indicating that the skyrmions motion occurs during the entirety of the pulse. In addition, the same result is obtained when we change the polarity of perpendicular field (see Supplementary Note 5). The obtained results indicate a formation of SyAF-coupled skyrmion bubbles between two ferromagnetic layers, which can be moved by much smaller current density than previously-studied ferromagnetic systems.

Now we elaborate mechanisms for the observed efficient motion of SyAF skyrmion bubbles. According to extended Thiele’s equation for SOT-driven skyrmion bubble motion, the velocity *υ*_Ave_ is expressed as^22,23^

1 $$\nu _{{\mathrm{Ave}}} = {\sqrt {\nu _x^2 + \nu _y^2}} = \frac{1}{{D\sqrt {g^2 + \alpha ^2} }}\chi J,$$

where *υ*_*x*_ = −*αD*/(Q*^2^ + *α*^2^*D*^2^), *υ*_*y*_ = Q*/(Q*^2^ + *α*^2^*D*^2^), and *g* = Q*/*D*. *χ* is the SOT efficiency parameter which is proportional to (*ћ*/2*e*)(*ξ*_SL_/*m*) with *ћ* the Dirac constant, *e* the electron charge, *ξ*_SL_ the SOT efficiency, and *m* the net areal magnetic moments. Also, Q* is the effective topological charge given by *Q*(*m*_Com_/*m*_S_) with *Q* being the topological charge defined with the Néel vector, *D* the dissipative tensor, *α* the Gilbert damping constant, and *g* the topological damping constant^22^. As can be seen in Fig. 2c, d, *v*_Ave_ for SyAF skyrmion bubbles shows nonlinear (virtually linear) behavior with respect to *J* below (above) *J* = 6.5 × 10^11^ A m^−2^. This means that, at around this *J*, skyrmion motion transits from creep/depinning to flow regimes. Linear dependence between *v*_Ave_ and *J* in larger current-density, i.e., flow, region is consistent with Eq. (1)^24^. In this framework, the achieved efficient control of SyAF skyrmion bubbles can be attributed to the following two factors. Firstly, SOTs induced by bottom Pt and top W should bring an additive effect, leading to an enhanced *χ*^25^. It has been known that Pt^11,26^ and W^27^ exhibit sizable spin Hall effect with the opposite sign. Therefore, the spins of the same direction are accumulated at each ferromagnetic layer under a current application. These spins act on the SyAF-coupled chiral Néel DWs moving in the same direction, resulting in a reduction of current density for the motion of skyrmion bubbles. This is contrasting to the case with widely-studied ferromagnetic skyrmion bubbles in multilayer systems, where use of materials exhibiting the spin Hall effect with the same sign for top and bottom layers is preferable to reduce required current density^28,29^. In addition, we note that the intermediate Ir layer also exhibits slight yet positive contribution to each ferromagnetic layer. A separate experiment reveals that SOT in stack 1 is larger by a factor of 1.5 than stack 2 (see Supplementary Note 3). In addition, small net areal magnetic moment in SyAF structure increases the effective field of SOT because *χ* is inversely proportional to *m*. According to the results shown in Fig. 1b, c, areal magnetic moment is different between the two systems by a factor of 3.9. Secondly, reduction of Q* due to the SyAF structure also contributes to the efficient motion through a suppression of topological damping. It is known that SOT forces gyrotropic motion on magnetic skyrmion owing to the topology, in addition to translational motion. This leads to the topological damping and causes a waste of driving force due to the momentum conservation^22^. In SyAF system, because of a reduction of the effective topological charge Q*, the topological damping is suppressed, resulting in an efficient action of driving force on the translational motion. Assuming a reported *α* in literatures, this factor is estimated to be about 1.0 (ref. ^30^)−1.8 (ref. ^31^). Consequently, the SyAF system is expected to be efficient in terms of low-current and fast skyrmion motion by a factor of about 5−10. This estimation roughly explains the observed results. We also note that while increased velocity of DW due to an exchange torque in a SyAF system was reported^32^, this driving force vanishes in skyrmion bubbles due to the circular symmetry in two-dimensional space^22^. Overall, the employed stack structure can not only stabilize SyAF-coupled Néel skyrmion bubbles due to the additive DMI, but also can drive them efficiently due to the additive SOT and reduced effective topological charge through RKKY interaction.

### Skyrmion Hall effect in SyAF skyrmion bubbles

**Fig. 3:**
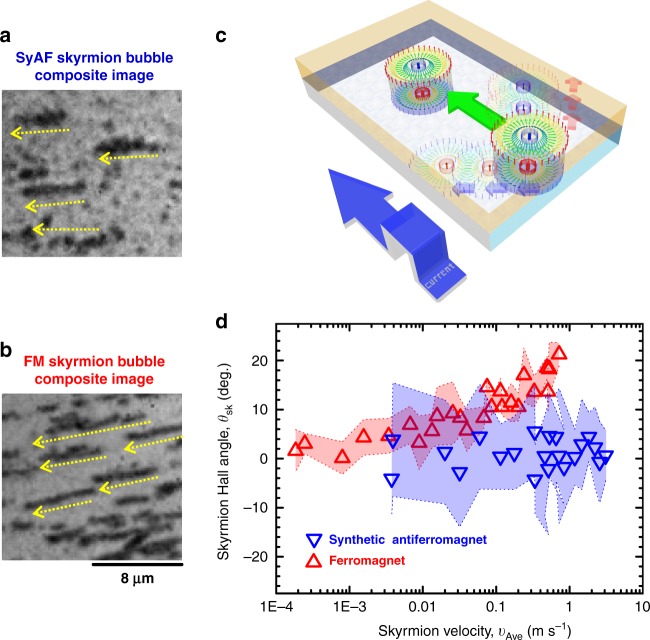
Skyrmion Hall effect in synthetic antiferromagnetic skyrmion bubbles vs ferromagnetic skyrmion bubbles. **a** The composite image of current-induced motion of SyAF skyrmion bubbles for three-pulses injection with the pulse width 1 μs. **b** The composite image of ferromagnetic skyrmion bubbles for three 5-μs-long pulses. Yellow arrows represent direction of displacement of skyrmion bubbles. **c** Schematic of the current-induced SyAF skyrmion motion in our experiment. **d** Skyrmion Hall angle as a function of average skyrmion velocity for synthetic antiferromagnetic (blue) and ferromagnetic (red) skyrmion bubbles. Colored range represents standard deviation

In addition to the advantage in terms of velocity and critical current density, the SyAF structure is expected to suppress SkHE due to the effectively compensated topological charge^16^. To examine it, we evaluate skyrmion Hall angle *θ*_sk_ ≡ tan^−1^(*l*_*y*_/*l*_*x*_) for current-induced motion of skyrmion bubbles, where *l*_*x*_ and *l*_*y*_ are displacement in the *x* and *y* directions (*x* direction is parallel to the current). Figure 3a, b show composite images (see Methods for details) of the magnetic states after sequential injection of three current pulses to stacks 1 and 2, respectively. It is clear that ferromagnetic skyrmion bubbles (Fig. 3b) move in the diagonal direction with respect to the current flow, indicating a sizable SkHE. On the other hand, for SyAF skyrmion bubbles, motion along the transverse direction is insignificant as expected (Fig. 3a) (see also Supplementary Movies and Supplementary Note 6 for the effect of compensation). Figure 3c shows a schematic of the SyAF skyrmion motion. In our system, skyrmion bubbles formed in the top (bottom) layer are subject to a Magnus force in the right (left) direction, eventually resulting in a motion of SyAF skyrmion along the current direction. We summarize the *θ*_sk_ as a function of average velocity of skyrmion bubbles in Fig. 3d. As for the ferromagnetic skyrmion bubbles, *θ*_sk_ increases monotonically with velocity. Such a velocity dependent *θ*_sk_ is consistent with previous studies that describe an effect of pinning^23^ and/or field-like torque^26^ on SkHE. Meanwhile, for SyAF skyrmion bubble, no noticeable *θ*_sk_ is observed, even in the depinning and flow regimes (*υ*_Ave_ > 0.1 m s^−1^ according to Fig. 2b) where sizable SkHE is reported in ferromagnetic systems. Such an inhibition of SkHE should be primarily caused by a reduction of the effective topological charge Q*. In addition, field-like torque-induced SkHE pointed out in a recent study^26^ should be also suppressed by the SyAF structure. We note that the material systems consisting of Pt/ferromagnet/Ir show relatively small SkHE compared to the other systems probably due to large *α* (see Supplementary Note 6). As shown above, SyAF skyrmion bubbles is promising to suppress the SkHE at room temperature, which has been a challenge of ferromagnetic and ferrimagnetic-alloy systems.

## Discussion

We have shown a formation of SyAF skyrmion bubbles at room temperature, which can be moved by SOT with negligible SkHE and much faster speed for a given current density than ferromagnetic systems. Hereafter, we discuss challenges and prospects of SyAF skyrmion bubbles based on the obtained results. Firstly, we elaborate on the velocity. While we have shown that SyAF skyrmion bubbles move faster than ferromagnetic counterpart, the achieved speed is smaller than that reported in a Pt/CoFeB/MgO multilayer^11^. This is presumably due to a smaller SOT and a larger depinning field in our systems (see Supplementary Note 3 and Note 7, respectively). In this regard, engineering of pinning as well as enhancement of SOT are important challenges to achieve even faster and lower-current operation. We also note that while SyAF skyrmion is free from the limitation of velocity caused by SkHE^33^, a micromagnetic simulation predicts another upper limit of accessible speed defined by the interlayer exchange coupling (see Supplementary Note 8). Secondly, the SyAF system is also beneficial in terms of formation of ultra-small skyrmions with high thermal stability. For ferromagnetic systems, small skyrmions (<10 nm) have been realized in single ultra-thin ferromagnetic layer only at low temperature due to low stability^5^, whereas multilayer systems achieves room temperature skyrmions with inevitably increased size due to a significant stray field^22^. Meanwhile, SyAF systems have a potential to accommodate ultra-small skyrmions at room temperature owing to an increased volume with negligible stray field. We also note that although compensated ferrimagnetic-alloy systems in principle share the advantage of the SyAF system, it is limited to a specific temperature due to different temperature dependence of angular momentum of the two sub-lattices.

In summary, this work shows a formation of SyAF-coupled skyrmion bubbles at room temperature. The emerged skyrmions can be driven by SOT with smaller current density and negligible SkHE than their ferromagnetic counterparts. Our findings will open a new avenue for future spintronic applications that achieves an energy-efficient and reliable electrical control of nanoscale skyrmions.

After the original submission of our manuscript, we learned that a similar work was reported by Legrand et al.^34^. They elaborate the formation of SyAF-coupled skyrmion, whereas we achieve a formation and motion of SyAF skyrmion by engineering the stack to effectively utilize DMI and SOT.

## Method

### Film preparation

The films were deposited at room temperature onto 3-inch thermally oxidized Si substrate. DC magnetron sputtering was used to deposit the layers. The base pressure of the chamber was less than 1 × 10^−6^ Pa and Ar gas was used for sputtering.

### Characterization of blanket film

The *m*–*H* loops were measured using a vibrating sample magnetometer, where *m* denotes the magnetic moment per unit area. Interlayer exchange field *H*_int_ was determined at the decreasing point of the *m*–*H*_*z*_ loop from the saturation state. Dzyaloshinskii-Moriya effective field *H*_DMI_ and domain wall chirality was evaluated by magnetic field-induced domain wall motion^35–38^ and current-induced magnetic hysteresis loop shift^39^ which can simultaneously evaluate spin-orbit torque contribution for each heavy metal.

### Device fabrication

The deposited films were processed into Hall bar devices by photolithography and Ar ion milling. The width and length of the channel of the devices were 50 and 100 µm, respectively. Electrodes made of Cr(5)/Au(100) were formed by photolithography and lift-off.

### Measurement of current-induced motion

All the measurements were performed at room temperature. Magnetic domain patterns were observed by MOKE with the wavelength of 542 nm. The differential image between uniform and non-uniform states was recorded to obtain better contrast. Image contrast was adjusted for each system; raw image of SyAF samples show weaker contrast than ferromagnetic samples. Current pulses with various pulse widths down to 500 ns were injected. Supplementary Movies were recorded with 61 frame per second. Skyrmion velocity and Hall angle were evaluated by an average of the motion under positive and negative current pulse applications for arbitrary-selected ten skyrmions; no skyrmions moving near pinned/nucleated/annihilated were included here. The composite image was created from three images, which were taken after a current pulse application, to make it easier to track each skyrmion bubble, as usually used for tracking the trajectory of stars in the night sky. Only the objects with relatively dark contrast were composited among images taken. We confirmed that drift is negligibly small compared with the observed motion of skyrmions. Error bars denote their standard deviation.

## Supplementary information

Supplementary Information

## Data Availability

The data which support the findings of this work are available from the corresponding author upon reasonable request.
