# Supplementary material for: Formation and current-induced motion of synthetic antiferromagnetic skyrmion bubbles
Source: Nat Commun. 2019 Nov 14;10:5153. doi: 10.1038/s41467-019-13182-6 (PMC6856122; doi:10.1038/s41467-019-13182-6)
Supplement: Supplementary file 1 — Supplementary Information [file 41467_2019_13182_MOESM1_ESM.pdf]

Formation and current-induced motion of synthetic antiferromagnetic skyrmion bubbles

Dohi *et al.*

SUPPLEMENTARY INFORMATION

Supplementary Notes:

1. Interlayer and ferromagnetic layer thickness dependence

2. Domain wall chirality evaluated by magnetic bubble expansion

3. Magnitude of Dzyaloshinskii-Moriya interaction and spin-orbit torque efficiency evaluated by current-induced magnetic hysteresis loop shift

4. Micromagnetic simulation for the size and topology of synthetic antiferromagnetic skyrmion bubble

5. Current pulse width and magnetic field polarity dependence of Synthetic antiferromagnetic skyrmion bubble motion

6. Current-induced elongation of synthetic antiferromagnetic skyrmion bubbles in Pt/Co/Ru/Co/W system for the effect of compensation

7. Difference in depinning field between Pt/Co/CoFeB/Ir and CoFeB/MgO systems

8. Velocity limit of synthetic antiferromagnetic skyrmion bubble derived by micromagnetic simulation

Supplementary Note 1

Interlayer and ferromagnetic layer thickness dependence

Ruderman-Kittel-Kasuya-Yosida (RKKY) interaction enables synthetic antiferromagnetic (SyAF) exchange coupling between two ferromagnets (FMs) with optimum interlayer thickness[1–5]. To maximize interlayer exchange coupling field H_{int} in our stacks at an optimum thickness, we investigate Ir thickness (t_{Ir}) dependence of m-H_z curve. Supplementary Figures 1a and 1c show stack structure with 0–1.0 nm and 1.1–1.4 nm of t_{Ir}, respectively. As shown in Supplementary Fig. 1b, saturation areal magnetic moment m_S decreases with increasing t_{Ir}, suggesting a decrease of the effective FM thickness. For t_{Ir} = 0–1.0 nm, any precipitous variation of magnetic moment associated with spin-flip magnetization process is not observed. Meanwhile, clear signatures of spin flip magnetization reversal are obtained for t_{Ir} = 1.1–1.4 nm (shown in Supplementary Fig. 1d), implying a synthetic antiferromagnetic coupling between the FM layers. The interlayer coupling field (H_{int}), represented by arrows in Supplementary Fig. 1d, attains a maximum at t_{Ir} = 1.3 nm. We speculate this maximum in H_{int} to correspond to the second maximum in RKKY oscillations. It is well known that the first maxima in RKKY interlayer coupling field would be attained at lower t_{Ir} ~ 0.4-0.6 nm[6,7]. However, a perpendicular easy axis cannot be obtained for t_{Ir} ~ 0.4-0.6 nm as observed from Supplementary Fig. 1b. The reason is probably related to the orientation of Ir on which top Co is placed. For thinner t_{Ir}, it seems to be difficult for Ir to obtain (111)-orientation where Co can archive perpendicular anisotropy at their interface[8]. Thus, our optimal t_{Ir} is 1.3 nm, where the largest interlayer antiferromagnetic coupling acts between two ferromagnets with perpendicular magnetic easy axis.

To obtain a fully compensated synthetic antiferromagnetic system, we inserted $[Co(0.3)/Ni(0.6)]_2$ structure between the Ir layer and the top ferromagnetic Co(0.3)/CoFeB

[Supplementary Fig. 1e]. Supplementary Figure 1f clearly shows almost fully compensated SyAF coupling ($m_{Com}/m_S \leq 5\%$) at the magnitude of $\mu_0 H_z$ less than 50–75 mT. Besides, we unambiguously observed characteristic hysteresis curve which implies the emergence of ferromagnetic skyrmion[9–11] around breaking point of SyAF coupling although we could not experimentally observe magnetic domain state owing to the limitation of magnetic field in our magneto-optical Kerr effect (MOKE) setup ($\mu_0 H_z \leq 20$ mT), which possibly connotes also the presence of multi-domain and/or skyrmion in the SyAF coupling state. Because our micromagnetic simulation reveals that the stack allows SyAF skyrmion to be stabilized in which also ferromagnetic skyrmion can be stabilized at high magnetic field breaking SyAF coupling [see Supplementary Note 4]. Importantly, our demonstration set an example that one easily can fully compensate magnetic moment between top and bottom magnet at room temperature that is a challenging for ferrimagnetic material systems.

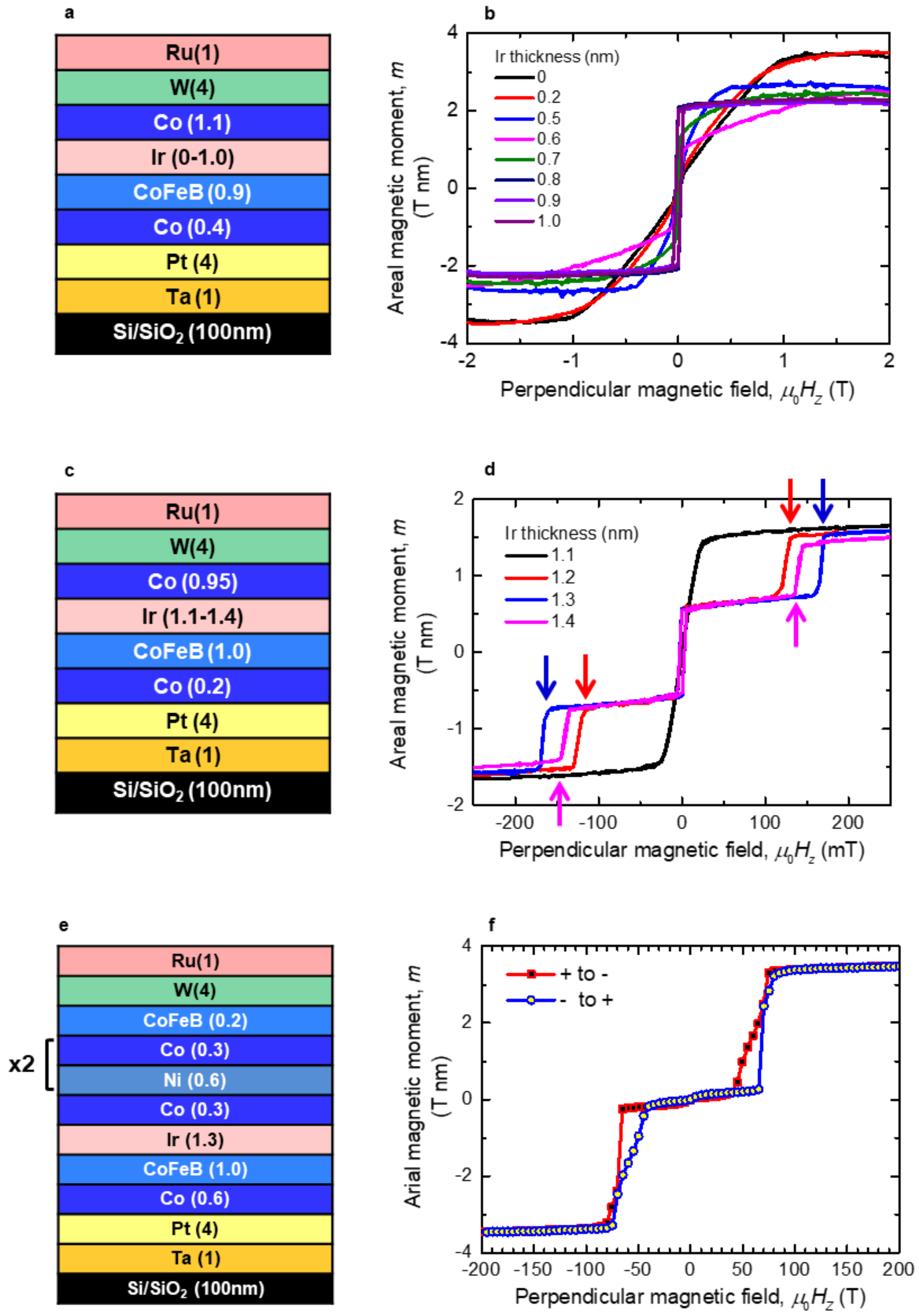

Supplementary Figure 1 | Ir and ferromagnetic layer thickness dependence of m-H_z curve. a, Stack structure used for range of 0-1.0 nm in Ir thickness and **b**, its m-H_z curve. **c**, Stack structure used for range of 1.1-1.4 nm in Ir thickness and **d**, its m-H_z curve. Colored arrow denotes H_{int} at each thickness. **e,** Stack structure used for realizing fully compensated magnetization state ($m_{Com}/m_S \leq 5\%$) and **f,** its m-H_z curve.

Supplementary Note 2

Domain wall chirality evaluated by magnetic bubble expansion

In this Note, we investigate Dzyaloshinskii-Moriya effective field H_{DMI} and domain wall (DW) chirality which governs current-induced motion of skyrmion bubble. Supplementary Figures 2a,d show stack structures used for the investigation of H_{DMI} which correspond to the bottom and top FMs, respectively, of the synthetic antiferromagnetic structure. We utilize asymmetric magnetic bubble expansion technique under simultaneous application of pulsed H_z and dc in-plane magnetic field H_x to evaluate DW chirality and H_{DMI} in the presence of interfacial Dzyaloshinskii-Moriya interaction (DMI)[12,13]. Starting from a saturated magnetic state, we first nucleate magnetic bubble by pulsed H_z in the absence of H_x. The magnetic bubble is then expanded under the simultaneous application of pulsed H_z and dc H_x. Supplementary Figure 2b shows magnetic bubble expansion under $\mu_0 H_x$ = ±104 mT for Pt/Co/CoFeB/Ir (bottom FM structure of the antiferromagnetic heterostructure). Asymmetric bubble expansion is observed, where the difference in DW velocity (υ_{DW}) arises from the modification of DW configuration at one side owing to the presence of H_x. We find that Pt/Co/CoFeB/Ir system exhibits left-handed/counter-clockwise chiral Néel DW corresponding to negative DMI, consistent with previous works[14–17]. Supplementary Figure 2c shows H_x dependence of υ_{DW} in Pt/Co/CoFeB/Ir system. A local minimum in υ_{DW}-H_x curve is obtained which corresponds to a maximum of DW energy due to energy balance between H_{DMI} and H_x. The minima in H_x equals H_{DMI}, when the antisymmetric contribution in υ_{DW} is small. For applied fields $\mu_0 H_z$ = 2.6 mT, υ_{DW}-H_x curve includes large antisymmetric contribution, hindering us to accurately evaluate H_{DMI} from this regime, as shown previously[18–20]. To remove the antisymmetric contribution from υ_{DW}, we increase pulse H_z beyond creep regime to quantify H_{DMI} from depinning and/or

flow regimes[21,22]. With increasing H_z, local minimum in υ_{DW} shifts towards the direction of larger H_x. This indicates that antisymmetric contribution leads to underestimation of H_{DMI}.

From υ_{DW}-H_x measurements under an applied $\mu_0 H_z$ = 7.5 mT, we determine the lower bound of $\mu_0 H_{DMI}$ to be ~80 mT for Pt/Co/CoFeB/Ir system. Application of larger H_z triggers multiple nucleation of magnetic domains hindering the evaluation of H_{DMI} by bubble expansion technique. Similar measurements on Ir/Co/CoFeB/W (top FM structure of the antiferromagnetic heterostructure) also exhibits an asymmetric behaviour in υ_{DW}, indicating a non-zero H_{DMI}, as can be seen in Supplementary Figs. 2e and 2f. In addition, the shift direction of local minimum in υ_{DW} with increasing pulse H_z is exactly the same to that for bottom FM, indicating left-handed/counter-clockwise DW chirality for top FM as well (local minimum in υ_{DW}-H_x shows left-handed/counter-clockwise chirality under $\mu_0 H_z$ = 11.6 mT)[22,23]. In summary, we obtain left-handed/counter-clockwise DW chirality for both Pt/Co/CoFeB/Ir and Ir/Co/CoFeB/W structures where, only lower bound concerning the magnitude could be determined by this method.

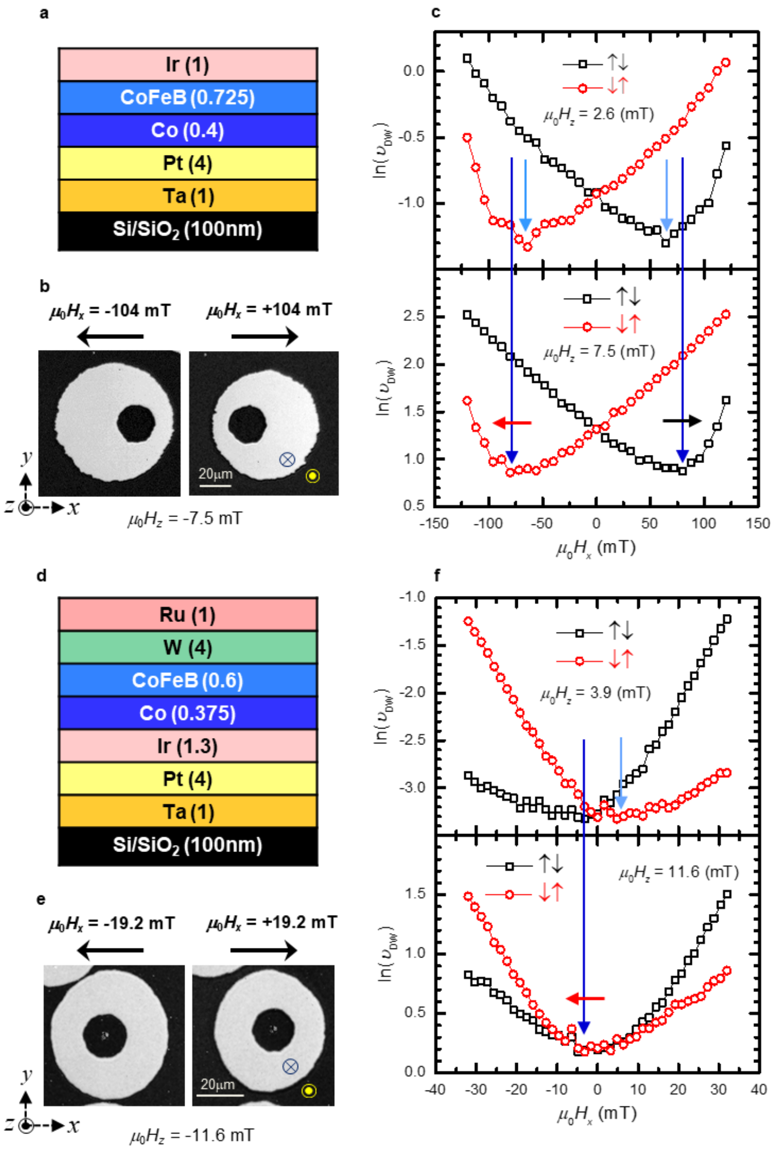

Supplementary Figure 2 | Evaluation of DW chirality and H_{DMI}. a, Stack structures used for evaluation in H_{DMI} and DW chirality for Pt/Co/CoFeB/Ir (bottom FM, hereafter), and **d**, Ir/Co/CoFeB/W (top FM, hereafter). **b**, Magnetic bubble expansion by $\mu_0 H_z$ of –7.5 mT under $\mu_0 H_x$ = ±104 mT for bottom FM, and **e**, by $\mu_0 H_z$ of –11.6 mT under $\mu_0 H_x$ = ±19.2 mT for top FM, where the arrow in image represents applied direction of H_x, ⊙, ⊗ denote up and down magnetic domain, respectively. **c**, $\mu_0 H_x$ dependence of υ_{DW} by $\mu_0 H_z$ of 2.6 mT, 7.5 mT for bottom FM, and **f**, by $\mu_0 H_z$ of 3.9 mT, 11.6 mT for top FM, where black and red color correspond to ↑↓ and ↓↑ DWs, white blue and blue arrows denote saddle point in each curve, sideways arrow mean shift direction of saddle point, respectively.

Supplementary Note 3
Magnitude of Dzyaloshinskii-Moriya interaction and spin-orbit torque efficiency evaluated by current-induced magnetic hysteresis loop shift

In this Note, we evaluate the magnitude of H_{DMI} and SOT efficiency χ_{SOT} by current-induced magnetic hysteresis loop shift. Supplementary Figure 3a shows a schematic of the experimental setup used in this Note. The effective spin-Hall-induced field ($H^{\mathrm{eff}}{}_z$) originating from the injection of current in these structures is expressed by

$$\mu_0 H_z^{\mathrm{eff}} = \chi_{\mathrm{SOT}} J \qquad (1)$$

where J is current density, χ_{SOT} corresponds to the SOT efficiency. For SOT-induced DW motion, χ_{SOT} depends on an angle θ of magnetic moment inside DW, and is modified as follows

$$\chi_{\mathrm{SOT}} = \chi(\cos\theta_{\mathrm{UP-DOWN}} + \cos\theta_{\mathrm{DOWN-UP}})/2 \qquad (2),$$

where UP-DOWN and DOWN-UP corresponds to DW from up to down and down to up with respect to x axis, respectively and χ denotes SOT parameter[24]. In the absence of any in-plane magnetic field, induced $\mu_0 H^{\mathrm{eff}}{}_z$ from in-plane current does not act effectively because of randomness of the angle or the fixed angle by DMI. As can be comprehended from Supplementary Note 2, since H_{DMI} compensates in-plane magnetic field, when in-plane magnetic field equals to H_{DMI}, χ_{SOT} can be saturated. For applied H_x equal H_{DMI}, magnetic moment inside DW aligns along H_x direction resulting in maximum χ_{SOT}. Thus, current-induced hysteresis loop shift measurements enable the simultaneous determination of H_{DMI} and χ_{SOT}.

Supplementary Figure 3b shows typical perpendicular anomalous Hall hysteresis loop with dc current I of 15 mA under in-plane magnetic field $\mu_0 H_x$ of 50 mT. We observe shift of anomalous Hall hysteresis loop by current, indicating that SOT act as $\mu_0 H^{\mathrm{eff}}{}_z$. Supplementary

Figure 3b shows linear proportionality between H^{eff}_z and current magnitude, in agreement with Supplementary Equation 1. χ_{SOT} is then obtained as a linear fit of H^{eff}_z vs current. Supplementary Figures 3d–3k show series of stack structures used to identify the amplitude of DMI and χ_{SOT} acting in different layers of synthetic antiferromagnet and H_x dependence of χ_{SOT}. The ratio of CoFeB thickness t_{CoFeB} and Co thickness t_{Co} is changed with fixed total thickness of the synthetic antiferromagnet to be $t_{\mathrm{CoFeB}} + t_{\mathrm{Co}}$, used in the main body. To determine current density J in each layer of the synthetic antiferromagnet structure, we measure sheet conductance as a function of thickness as shown in Supplementary Fig. 3p by using stack structure as shown in Supplementary Table 1. The saturation of χ_{SOT} is observed in all the series with large H_x. We find that saturated χ_{SOT} shows ferromagnetic thickness ratio independence.

Supplementary Table 2 shows the averaged magnitude of χ_{SOT} for different heavy metal layers, where error bar is derived from standard deviation of values obtained in same series. We find that major contribution to χ_{SOT} arises from Pt and W layers while that for Ir is much smaller, consistent with a previous work[25]. In whole system, the sign of χ_{SOT} is tailored to positive. This unified sign of χ_{SOT} at bottom and top HM layers assists in skyrmion bubble motion. While, we comprehended that these amplitudes are one-order smaller than those of ferromagnetic systems tailored by multiple repetition of FM/HM heterostructure[26,27], presumably leading to significant difference in the FM skyrmion bubble velocity between such systems[9] and our systems.

The amplitude of interfacial DMI D_{i} is obtained from[14,28,29]

$$D_{\mathrm{i}} = M_{\mathrm{S}} H_{\mathrm{DMI}} \sqrt{A_{\mathrm{S}} / K_{\mathrm{eff}}} \quad (3),$$

where H_{DMI} equals H_x at which χ_{SOT} attains saturation, M_{S} is spontaneous magnetization, A_{S} is exchange stiffness and K_{eff} is effective magnetic anisotropy energy density. M_{S} and K_{eff} are

experimentally determined from in-plane and out-of-plane magnetization curves. A_S is assumed to be 8 pJ m^{-1}, typical for CoFeB[30–32]. The sign of D_i is deduced from magnetic bubble expansion measurements in Supplementary Note 2. Supplementary Figures 3l–3o show the Co and CoFeB layer thickness ratio dependence of D_i. D_i for single ferromagnet, Co(CoFeB) is extracted from extrapolation of t_{CoFeB}/t_{Co}(t_{CoFeB}/t_{Co}). Supplementary Table 3 indicates the obtained magnitude of D_i from all the series. We find that Pt and W exhibits significant contribution to DMI in our stack structures. In addition, as shown in Supplementary Fig. 3m, extrapolation of D_i versus t_{Co}/t_{CoFeB} to y-axis in Ir/Co/CoFeB/Ru stacks means positive or zero DMI, indicating a contribution of DMI from Ir in these synthetic antiferromagnet due to non-cancellation at CoFeB/Ir (bottom side) and Ir/Co (top side) interfaces. The evaluation of DMI by the extrapolation results in a value of D_i = –0.20 mJ m^{-2} and –0.55 mJ m^{-2} for top structure and bottom structure by using t_{Co}/t_{CoFeB} = 0.625 and 0.21, respectively. In Supplementary Note 4, we utilize the obtained magnitudes of D_i to verify topological protection and static size of SyAF skyrmion bubble in our stacks by micromagnetic simulation.

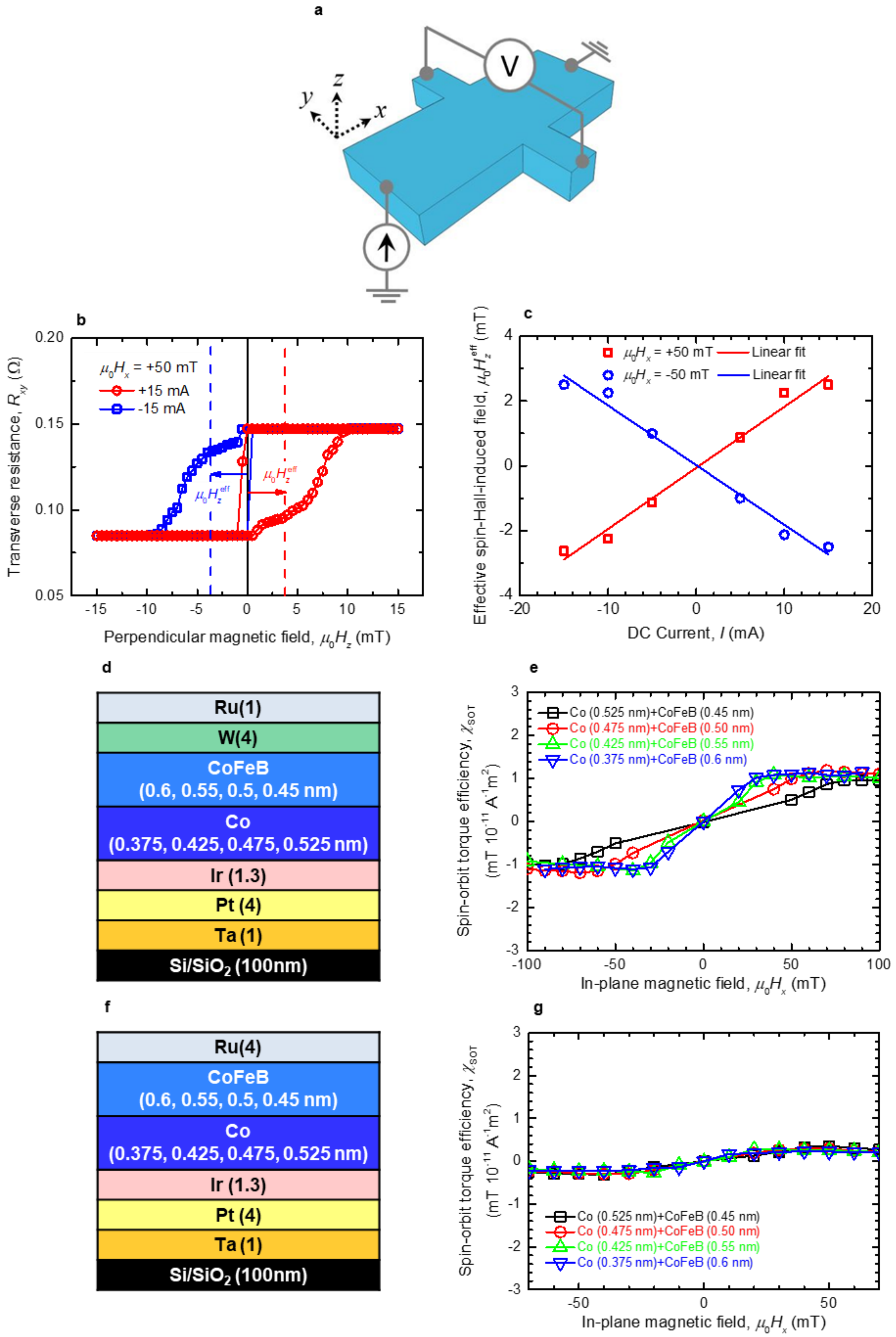

a
V
y
z
x
b
Transverse resistance, R_{xy} (Ω)
μ_0H_x = +50 mT
+15 mA
-15 mA
$\mu_0H_z^{eff}$
Perpendicular magnetic field, μ_0H_z (mT)
c
Effective spin-Hall-induced field, $\mu_0H_z^{eff}$ (mT)
μ_0H_x = +50 mT
μ_0H_x = -50 mT
Linear fit
DC Current, I (mA)
d
Ru(1)
W(4)
CoFeB (0.6, 0.55, 0.5, 0.45 nm)
Co (0.375, 0.425, 0.475, 0.525 nm)
Ir (1.3)
Pt (4)
Ta (1)
Si/SiO_2 (100nm)
e
Spin-orbit torque efficiency, χ_{SOT} (mT 10^{-11} $A^{-1}m^2$)
Co (0.525 nm)+CoFeB (0.45 nm)
Co (0.475 nm)+CoFeB (0.50 nm)
Co (0.425 nm)+CoFeB (0.55 nm)
Co (0.375 nm)+CoFeB (0.6 nm)
In-plane magnetic field, μ_0H_x (mT)
f
Ru(4)
CoFeB (0.6, 0.55, 0.5, 0.45 nm)
Co (0.375, 0.425, 0.475, 0.525 nm)
Ir (1.3)
Pt (4)
Ta (1)
Si/SiO_2 (100nm)
g
Spin-orbit torque efficiency, χ_{SOT} (mT 10^{-11} $A^{-1}m^2$)
Co (0.525 nm)+CoFeB (0.45 nm)
Co (0.475 nm)+CoFeB (0.50 nm)
Co (0.425 nm)+CoFeB (0.55 nm)
Co (0.375 nm)+CoFeB (0.6 nm)
In-plane magnetic field, μ_0H_x (mT)

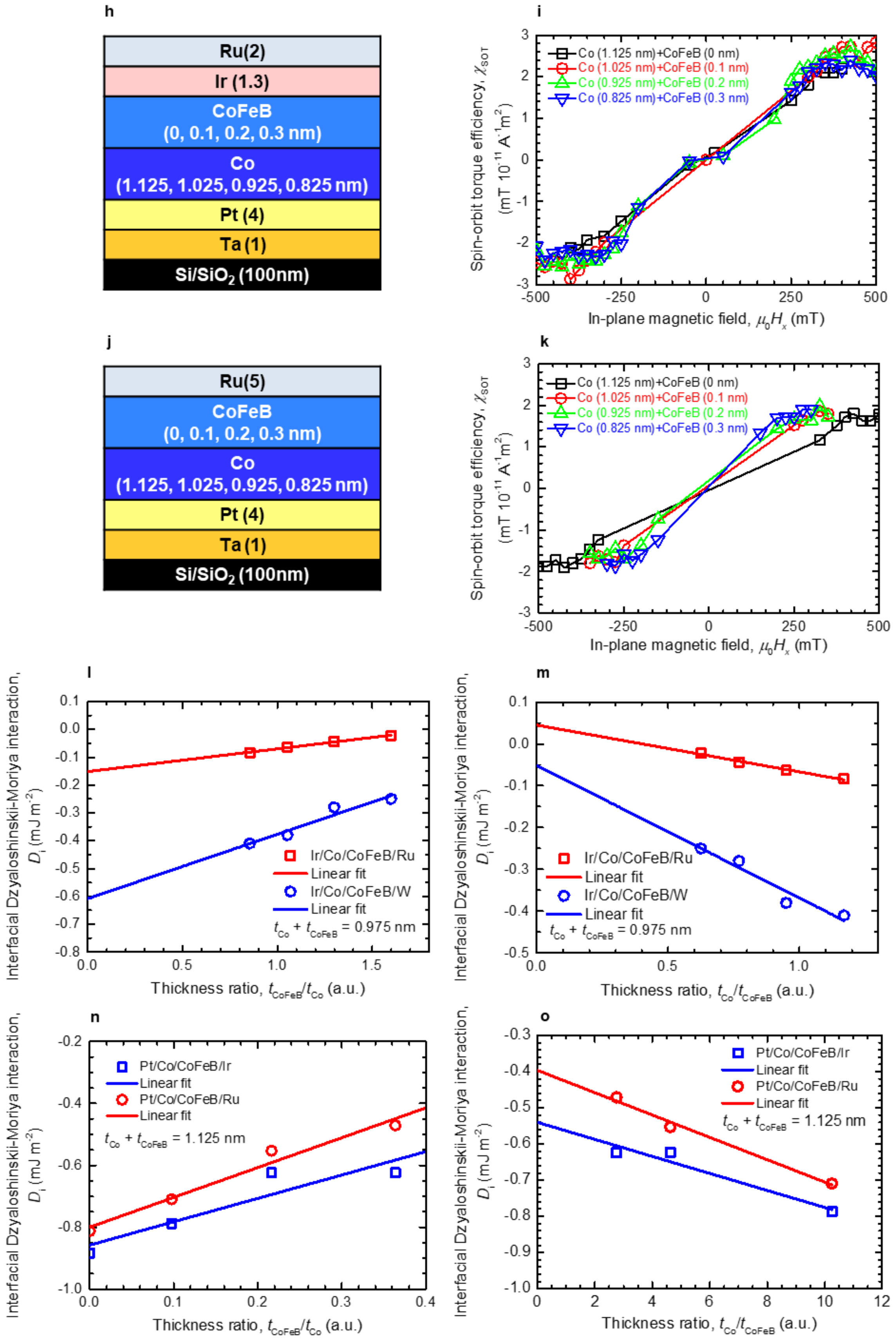
h
Ru(2)
Ir (1.3)
CoFeB
(0, 0.1, 0.2, 0.3 nm)
Co
(1.125, 1.025, 0.925, 0.825 nm)
Pt (4)
Ta (1)
Si/SiO2 (100nm)
i
Co (1.125 nm)+CoFeB (0 nm)
Co (1.025 nm)+CoFeB (0.1 nm)
Co (0.925 nm)+CoFeB (0.2 nm)
Co (0.825 nm)+CoFeB (0.3 nm)
Spin-orbit torque efficiency, χSOT (mT 10-11 A-1m2)
In-plane magnetic field, μ0Hx (mT)
j
Ru(5)
CoFeB
(0, 0.1, 0.2, 0.3 nm)
Co
(1.125, 1.025, 0.925, 0.825 nm)
Pt (4)
Ta (1)
Si/SiO2 (100nm)
k
Co (1.125 nm)+CoFeB (0 nm)
Co (1.025 nm)+CoFeB (0.1 nm)
Co (0.925 nm)+CoFeB (0.2 nm)
Co (0.825 nm)+CoFeB (0.3 nm)
Spin-orbit torque efficiency, χSOT (mT 10-11 A-1m2)
In-plane magnetic field, μ0Hx (mT)
l
Ir/Co/CoFeB/Ru
Linear fit
Ir/Co/CoFeB/W
Linear fit
tCo + tCoFeB = 0.975 nm
Interfacial Dzyaloshinskii-Moriya interaction, Di (mJ m-2)
Thickness ratio, tCoFeB/tCo (a.u.)
m
Ir/Co/CoFeB/Ru
Linear fit
Ir/Co/CoFeB/W
Linear fit
tCo + tCoFeB = 0.975 nm
Interfacial Dzyaloshinskii-Moriya interaction, Di (mJ m-2)
Thickness ratio, tCo/tCoFeB (a.u.)
n
Pt/Co/CoFeB/Ir
Linear fit
Pt/Co/CoFeB/Ru
Linear fit
tCo + tCoFeB = 1.125 nm
Interfacial Dzyaloshinskii-Moriya interaction, Di (mJ m-2)
Thickness ratio, tCoFeB/tCo (a.u.)
o
Pt/Co/CoFeB/Ir
Linear fit
Pt/Co/CoFeB/Ru
Linear fit
tCo + tCoFeB = 1.125 nm
Interfacial Dzyaloshinskii-Moriya interaction, Di (mJ m-2)
Thickness ratio, tCo/tCoFeB (a.u.)

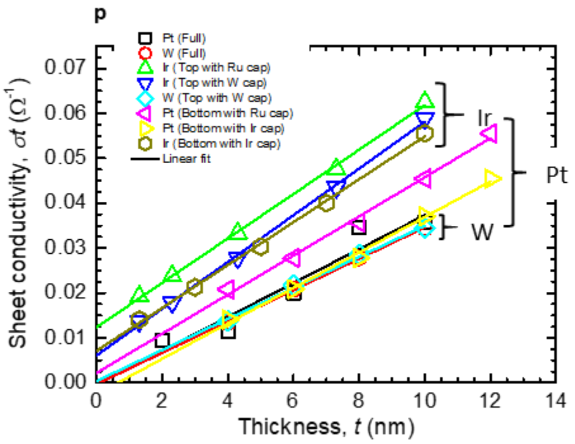

Supplementary Figure 3 | Evaluation of H_{DMI} and χ_{SOT}. **a**, Schematic of experimental setup. **b**, Typical H_z dependence of transverse resistance with $|I|$ = 15 mA under $\mu_0 H_x$ = 50 mT for the stack structure consisting of Si/SiO_2/Ta/Pt/Ir/Co(0.60)/CoFeB(0.375)/W/Ru where the units in parenthesis denotes nm, red and blue means plus current and minus current, respectively. **c**, Typical the current amplitude dependence of $\mu_0 H^{eff}{}_z$ in stack using for Fig. S3b where solid line means each linear fit. **d, f, h** and **j,** Series of stack structures used to reveal contribution to DMI and SOT every layer. **e,** H_x dependence of for the series in Fig. S3d, **g,** for the series in Fig. S3f, **i,** for the series in Fig. S3h and **k,** for the series in Fig. S3j. **l,** Thickness ratio of $t_{CoFeB}/t_{Co,}$ and **m,** t_{Co}/t_{CoFeB} dependence of D_i with keeping total thickness (t_{Co} + t_{CoFeB} = 0.975 nm) to be constant, where blue, red color and solid line represent for the series in Fig. S3d, Fig. S3f and their linear fit. **n,** Thickness ratio of $t_{CoFeB}/t_{Co,}$ and **o,** t_{Co}/t_{CoFeB} dependence of D_i with keeping total thickness (t_{Co} + t_{CoFeB} = 1.125 nm) to be constant where blue, red color and solid line represent for the series in Fig. S3h, Fig. S3j and their linear fit. **p,** Thickness dependence of sheet conductivity for the series of stack structures in Table 1.

Supplementary Table 1 | Stack structure used for sheet conductivity measurement and obtained resistivity

Correspondence stack	Stack structure (nm)	Resistivity (μΩ cm)
Full Structure	Ta/Pt(2-10)/Co/CoFeB/Ir/Co/CoFeB/W/Ru	28.7 (W)
	Ta/Pt/Co/CoFeB/Ir/Co/CoFeB/W(4-10)/Ru	26.5 (Pt)
Top Structure	Ta/Pt/Ir(1.3-10.3)/Co/CoFeB/Ru	20.2 (Ir)
	Ta/Pt/Ir(1.3-10.3)/Co/CoFeB/W/Ru	19.2 (Ir)
	Ta/Pt/Ir/Co/CoFeB/W(4-10)/Ru	28.9 (W)
Bottom Structure	Ta/Pt(4-10)/Co/CoFeB/Ru	23.0 (Pt)
	Ta/Pt(4-10)/Co/CoFeB/Ir/Ru	25.3 (Pt)
	Ta/Pt/Co/CoFeB/Ir(1-10)/Ru	21.0 (Ir)

Supplementary Table 2 | Spin-orbit torque efficiency χ_{SOT} for each heavy metal layer

Correspondence stack	Materials	Spin-orbit torque efficiency χ_{SOT} (mT 10^{-11} $A^{-1}m^2$)
Top Structure	Ir + W	+1.07 (±0.08)
	Ir	+0.25 (±0.05)
Bottom Structure	Pt + Ir	+2.34 (±0.21)
	Pt	+1.74 (±0.11)

Supplementary Table 3 | Interfacial DMI D_i for each stack structure

Correspondence stack	Structure	D_i (mJ m^{-2})
Top Structure (t = 0.975 nm)	Ir/CoFeB/Ru	+0.04 (±0.01) or 0
	Ir/CoFeB/W	-0.05 (±0.05)
	Ir/Co/Ru	-0.15 (±0.06)
	Ir/Co/W	-0.61 (±0.05)
Bottom Structure (t = 1.125 nm)	Pt/CoFeB/Ru	-0.40 (±0.02)
	Pt/CoFeB/Ir	-0.54 (±0.04)
	Pt/Co/Ru	-0.80 (±0.03)
	Pt/Co/Ir	-0.85 (±0.05)

Supplementary Note 4

Micromagnetic simulation for the size and topology of synthetic antiferromagnetic skyrmion bubble

We utilize micromagnetic simulation to examine topological protection and sizes of SyAF skyrmion bubble in our stack structures. We use MuMax3 as micromagnetic simulation software[33]. Supplementary Table 4 displays the parameter list used for simulation. To avoid from divergence of calculation time, an effective medium model is employed[9], where in magnetization compensation ratio $m_{\mathrm{Com}}/m_{\mathrm{S}}$ is controlled by directly adjusting spontaneous magnetization M_{S}. The other parameters are also modified accordingly. Starting from an antiferromagnetic checkerboard structure (Supplementary Fig. 4a), the system is relaxed to explore energetically stable state[9].

First, we carry out simulation with interfacial DMI D_{i1} and $D_{\mathrm{i2}} = 0$ and interlayer exchange coupling $J_{\mathrm{int}} = 0$. Supplementary Figure 4b shows the *x, y* and *z* components of normalized magnetization obtained after relaxation of the system from initial configuration. As expected, *z* component in top layer and magnified *x* component indicate a topologically trivial magnetic bubble without any synthetic antiferromagnetic coupling.

We then, slightly increased J_{int} to -0.05 mJ m^{-2} which is less than half the experimental value. The relaxed magnetic configuration (Supplementary Fig. 4c) shows SyAF magnetic bubble without any topological protection, possibly originating from the absence of DMI. The stabilization of magnetic bubbles from micromagnetic simulations for much smaller J_{int} than that observed from experiments justifies the realization of SyAF coupled magnetic domain in our antiferromagnetic stacks.

Finally, we utilize experimentally obtained magnetic parameters (shown in Supplementary Table 4) and investigate the possibility of realization of topologically non-trivial skyrmion bubble with SyAF coupling in these structures. Our simulation results, shown in Supplementary Fig. 4d, reveals the stabilization of topologically protected SyAF skyrmion bubble with left-handed DW chirality. Further simulations by varying A_S and M_{S1} up to 20 pJ m^{-1} and from 1.2 to 1.7 T, respectively, results in subtle modulation of size keeping other properties invariant. Supplementary Figure 4e shows magnetic field (H_z) dependence of the z-component of magnetization. For $\mu_0 H_z$ = 0 mT, SyAF multi-domain state is stabilized. Then, SyAF skyrmion bubble emerges with increasing H_z. For $\mu_0 H_z \geq$ 150 mT, antiferromagnetic interlayer exchange coupling is broken resulting in creation of ferromagnetic skyrmion bubble. Finally, ferromagnetic uniform state is stabilized for $\mu_0 H_z$ = 200 mT. The obtained behaviour from simulations are in good agreement with our experimental results, indicating feasible SyAF skyrmion state with the experimentally obtained parameters in Supplementary Table 4.

To further prove the realization of SyAF skyrmion in our structures and possible emergence of any ferromagnetic skyrmion state, we investigate the initial magnetization configuration dependence of skyrmions as follows:

#1. Bottom : Uniform (−), Top : Checkerboard

#2. Bottom : Uniform (+), Top : Checkerboard

#3. Bottom : Checkerboard, Top : Uniform (−)

#4. Bottom : Checkerboard, Top : Uniform (+)

#5. Bottom and Top : Checkerboard (ferromagnetic configuration, meaning that top and bottom layers possess completely same array),

where the sign in parenthesis denotes the direction of *z*-component of magnetization. #1, #2, #3 and #4 configurations result in a stable antiferromagnet uniform state while #5 relaxes to SyAF skyrmion bubble state. The present results validate the absence of any stable/quasi-stable ferromagnetic skyrmion state under applied magnetic field in our stack structures.

Finally, we roughly evaluate the size of the SyAF skyrmion bubble by changing checkerboard size at the initial state. Supplementary Table 5 summarizes initial checkerboard size versus the obtained size of SyAF skyrmion bubble at the converged state. Our simulation results suggest the presence of energy potential well with the size of 1 to 2 μm, which is roughly consistent with experimental observation in Supplementary Fig. 4f, where the skyrmion bubble diameter D_{sk} in SyAF skyrmion bubbles are determined from total no. of pixels within yellow rectangle for various H_z. In addition, the result shows that the size of these skyrmion bubbles is insensitive to the magnetic field as long as bubble structure can be formed, which is also consistent with micromagnetic simulation. We note that further scaling of skyrmion sizes down to technologically relevant sizes of a few tens of nano-meters requires tuning of the various parameters such as K_{eff}, D_i and J_{int}.

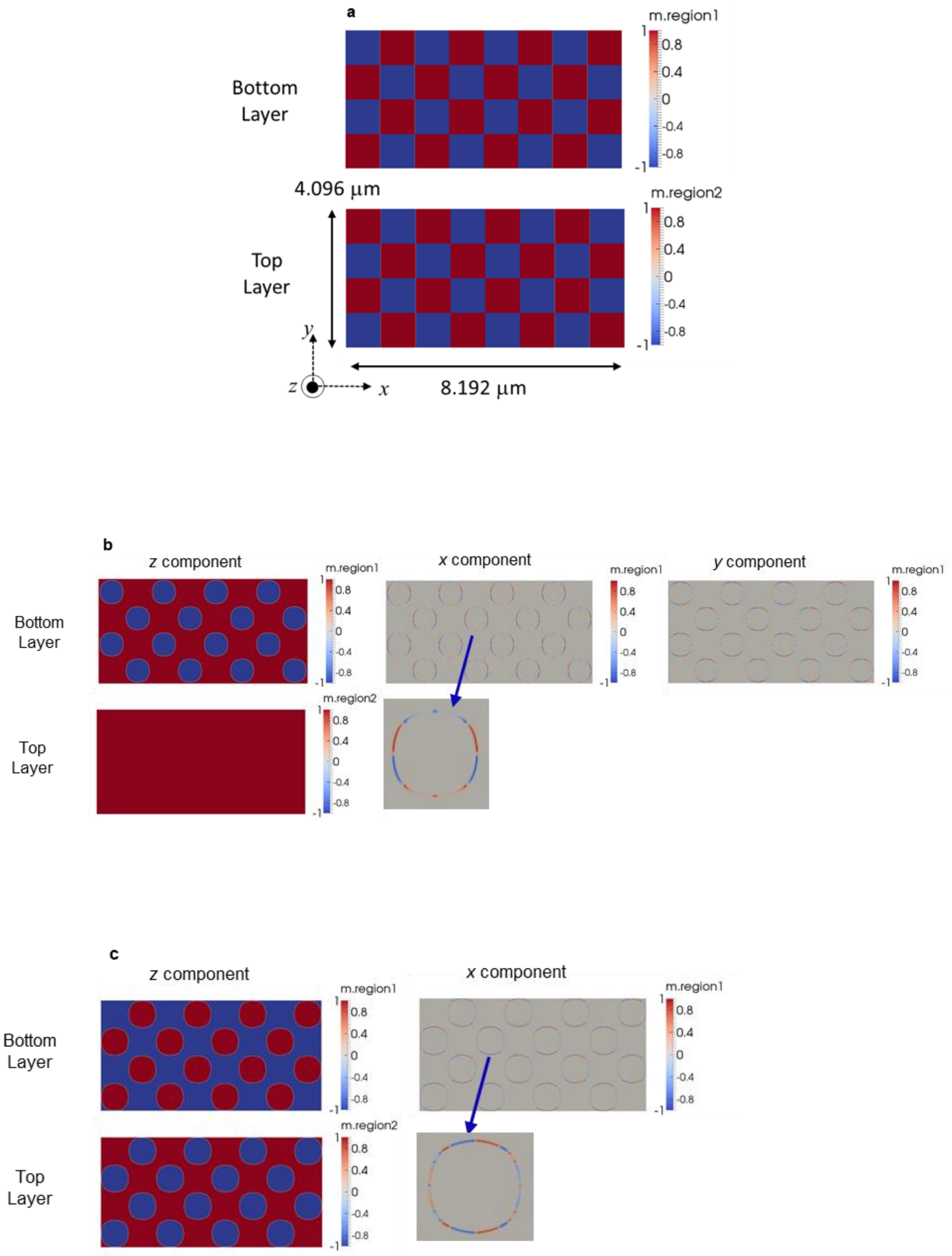

a
m.region1
Bottom Layer
4.096 μm
m.region2
Top Layer
8.192 μm
b
z component
x component
y component
c
z component
x component

d

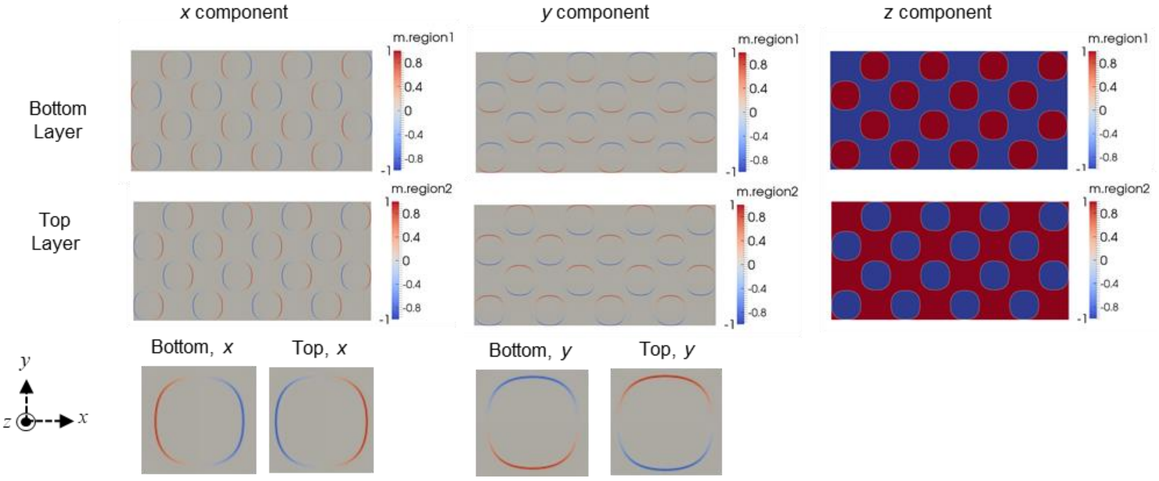
x component
y component
z component
Bottom Layer
Top Layer
m.region1
m.region2
0.8
0.4
0
-0.4
-0.8
1
-1
Bottom, x
Top, x
Bottom, y
Top, y
y
z
x

e

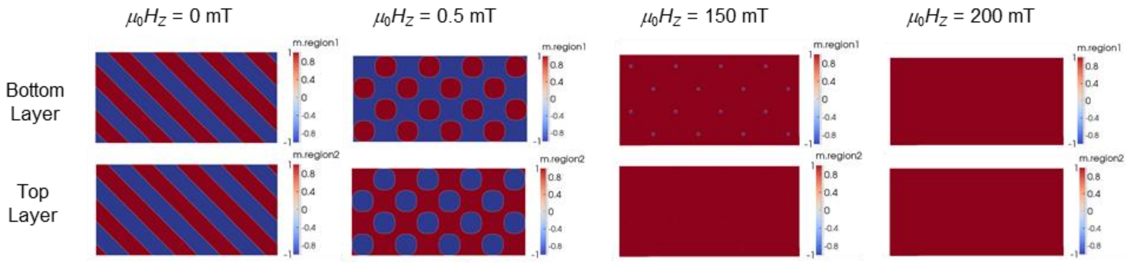
μ_0H_Z = 0 mT
μ_0H_Z = 0.5 mT
μ_0H_Z = 150 mT
μ_0H_Z = 200 mT
Bottom Layer
Top Layer
m.region1
m.region2

f

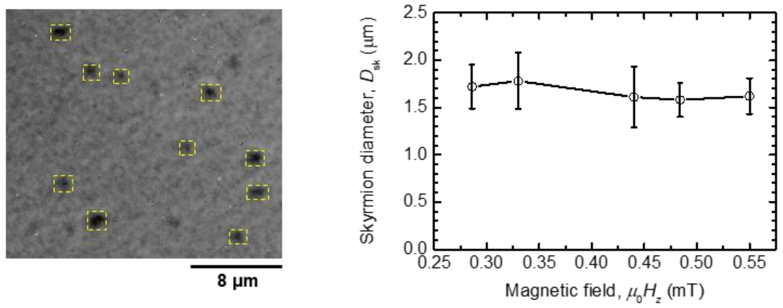
8 μm
Skyrmion diameter, D_{sk} (μm)
Magnetic field, μ_0H_z (mT)
2.5
2.0
1.5
1.0
0.5
0.0
0.25
0.30
0.35
0.40
0.45
0.50
0.55

Supplementary Figure 4 | Validation of topology and stabilizing SyAF skyrmion bubble. a, Initial state of antiferromagnetic checker board without any in-plane component of magnetic moment, where red and blue color represent up and down spin, respectively. **b**, The magnetic state after relaxation for using $D_{i1,\ i2} = 0$, $J_{int} = 0$, *z* component, *x* component and *y* component of spin are placed on left, center (with the magnified one) and right, respectively. All the components are re-normalized. **c**, The magnetic state after relaxation for using $D_{i1,\ i2} = 0$, $J_{int} = -0.05$ mJ m^{-2}, *z* component and *x* component of spin are placed on left and center (with the magnified one), respectively. **d,** The magnetic state after relaxation for using parameter in Table 4, *z* component, *x* component and *y* component of spin are placed on left, center (with the magnified one) and right, respectively. **e,** Magnetic field dependence of *z* component of spin for using parameter in Table 4. The results at $\mu_0 H_z = 0$, 0.5, 150 and 200 mT are on display from left side. **f,** The typical example of the picture used for determining the diameter of SyAF skyrmion bubble D_{sk} (left) is derived from total number of pixels within yellow rectangular shape and the magnetic field dependence of D_{sk}.

Supplementary Table 4 | Micromagnetic simulation parameter list for validation of size and topology of the synthetic antiferromagnetic skyrmion bubble

World size (top, bottom thickness)	8192x4096x2 nm^3 (1 nm, 1 nm)
Cell size	4x4x1, (2x2x1) nm^3
Periodic boundary condition	4x4x0
Spontaneous magnetization, M_S	1.5, 0.8 T
Uniaxial anisotropy energy density, K_{U1}, K_{U2}	1.01, 0.46 MJ m^{-3}
Interfacial DMI, D_{i1}, D_{i2}	-0.55, -0.2 mJ m^{-2}
Exchange stiffness, A_S	8 pJ m^{-1}
Interlayer exchange coupling J_{int}	-0.13 mJ m^{-2}
Gilbert damping, α	0.3
External perpendicular magnetic field $\mu_0 H_Z$	0.5 mT

Supplementary Table 5 | Number of cells and stable diameter of the SyAF skyrmion bubble

No. of cells along *x* and *y* (Cell*x* X Cell*y*)	Stable or not	Diameter
32 x 16	Not stable	N/A
16 x 8	Not stable	N/A
12 x 6	Not stable	N/A
8 x 4	Stable	1.04 μm
4 x 2	Stable	2.14 μm

Supplementary Note 5

Current pulse width and magnetic field polarity dependence of Synthetic antiferromagnetic skyrmion bubble motion

Here, we study pulse width dependence of velocity of SyAF skyrmion bubble in our structures. Supplementary Figure 5 shows current pulse width dependence of SyAF skyrmion bubble displacement as a function of current density J. A near-linear dependence of skyrmion displacement is observed within the entire range of J used in this study (Supplementary Figs. 5a-c). As can be seen, we observe linear behavior of skyrmion displacement in the studied range of pulse amplitude and width.

In addition to that, we also confirm different magnetic polarity dependence of averaged skyrmion velocity υ_{Ave}. Supplementary Figure 5d show that skyrmion velocity υ_{Ave} vs J with added plots represented by green color. We find that added plots unambiguously traces the trend shown in main body. This fact indicates that any extrinsic effects such as Oersted field and remanent field inherent to the measurement system does not influence the obtained results.

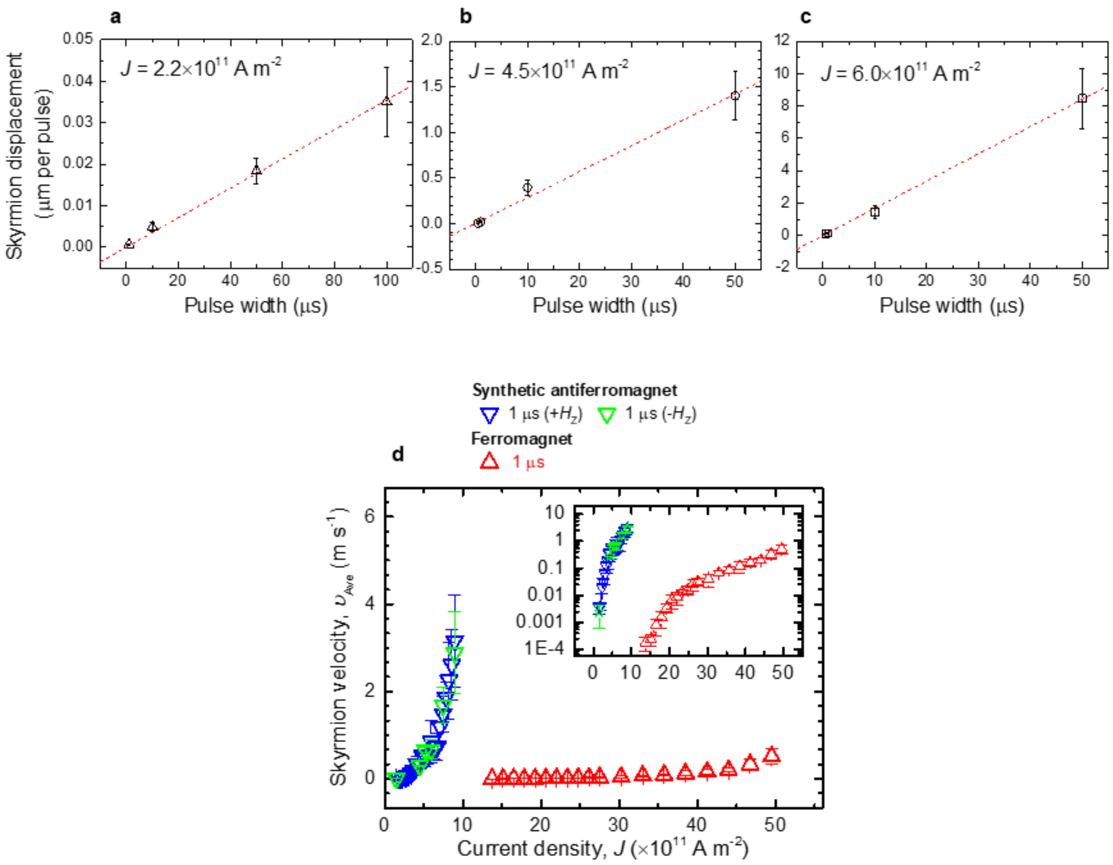

Supplementary Figure 5 | Current pulse width and different magnetic polarity dependence of SyAF skyrmion bubble displacement. a, Skyrmion displacement per pulse as a function of pulse width at current density J = 2.2×10^{11} A m^{-2}, **b**, at J = 4.5×10^{11} A m^{-2} and **c**, at J = 6.0×10^{11} A m^{-2}. Red broken line is guide to eye demonstrating the linear relationship. **d,** Skyrmion velocity vs J for perpendicular magnetic field $\mu_0 H_Z$ of +0.2 mT (blue) and -0.2 mT (green).

Supplementary Note 6

Current-induced elongation of synthetic antiferromagnetic skyrmion bubbles in Pt/Co/Ru/Co/W system for the effect of compensation

To capture more clearly the impact of magnetic moment compensation on skyrmion Hall effect (SkHE), we investigate current-induced elongation of synthetic antiferromagnetic skyrmion bubbles meaning the motion of half-skyrmion bubbles[40] by using Pt/Co/Ru/Co/W system, where a strong pinning potential is expected[35]. Supplementary Figures 6a and 6d show stack structures for nominal FM thickness ratio = 1 (stack S1, hereafter) and for different nominal FM thickness ratio (stack S2, hereafter), respectively. As can be seen in Supplementary Figs. 6b and 6e, each m-H_z curve unambiguously indicate SyAF coupling as compared to the stack without Ru layer (both stacks experience no saturation up to $\mu_0 H$ = 2 T for m-H curve for both in-plane and perpendicular direction probably due to large perpendicular magnetic anisotropy and interlayer exchange coupling). We determine the magnetization compensation degree m_{Com}/m_S (m_{Com} refers to areal magnetic moment at compensated state, m_S refers to spontaneous areal magnetic moment obtained from stack without Ru layer) to be 0.20 for stack S1 and to be 0.47 for stack S2, respectively. As shown in Supplementary Figs. 6c and 6f, we observe SyAF skyrmion bubble in stacks S1 and S2, as well as for Pt/Co/CoFeB/Ir/Co/CoFeB/W structure (shown in the main body).

Current-induced elongation of SyAF skyrmion bubble in stack S1 is observed in Supplementary Movies 3, 4, 5, 6 corresponding to the situation for negative current pulse $-J$ under $+H_z$, for $+J$ under $+H_z$, for $-J$ under $-H_z$ and for $+J$ under $-H_z$, respectively, where pulse width, current density $|J|$ and the magnitude of H_z are 1 ms, 14.4×10^{11} A m^{-2} and 12 mT, respectively. Supplementary Figure 6g summarizes symmetry for current-induced elongation of SyAF skyrmion bubbles. As can be seen in Supplementary Fig. 6g, current-dependent

asymmetric pinning is observed, which means SyAF skyrmion bubble gets elongated along the current flow direction with a finite angle. We note that this elongation cannot stem from trivial topology as magnetic bubble should be annihilated by injecting either +*J* or –*J* [41]. One of the possible reasons behind this peculiar behavior could be related to strong pinning potential in Pt/Co/Ru/Co/Pt systems as skyrmion is usually nucleated in pinning site acting as topological defect[42] except for relying on local stimuli[43,44], hence skyrmion seems to be bound tightly to there with large pinning potential. Secondly, we comprehend that SOT is a predominant factor for the current-induced elongation and stabilization of Néel-type SyAF skyrmion bubble as mentioned before (in the main text). Thirdly, we also distinctly capture the emergence of skyrmion Hall effect where their topological protection is identified from the trajectory of diagonal elongation of SyAF skyrmion bubbles. The symmetry is also consistent with the scenario described by left-handed/counter-clockwise DW chirality with positive spin Hall angle as well as Ir case. Supplementary Figures 6h and 6i show magneto-optical Kerr images captured after nucleation of SyAF skyrmion bubbles and injecting current pulse for stack S1 and stack S2, respectively. The modified diagonal elongation is recognized unambiguously between these two stacks, where more compensated SyAF skyrmion bubble (in stack S1) possesses smaller skyrmion Hall angle θ_{sk} as compared to that in stack S2. This is ascribed to decreasing effective topological charge as expected from main body. We also find that all the diagonal elongation ($-J$ under $+H_z$, $+J$ under $+H_z$, $-J$ under $-H_z$ and $+J$ under $-H_z$) presents almost the same magnitude of θ_{sk} as shown in Supplementary Fig. 6j and 6k, which bolsters up that SyAF skyrmion bubbles are non-trivial magnetic objects in this system. θ_{sk} in Pt/Co/Ru/Co/Pt system is larger than that for Pt/Co/CoFeB/Ir/Co/CoFeB/W system despite similar magnetization compensation ratio, which would arise from the difference in effective Gilbert damping constant α. In general, α tends to increase with the presence of heavy metal

owing to increasing spin-mixing conductance[16,45]. The diagonal motion of skyrmion is inversely proportional to α ($\upsilon_y/\upsilon_x = -Q^*/\alpha D$, as described in the main body). Thus, the presence of light element such as Ru in the neighbourhood of FM would explain well observed rather larger θ_{sk} due to decreasing averaged α.

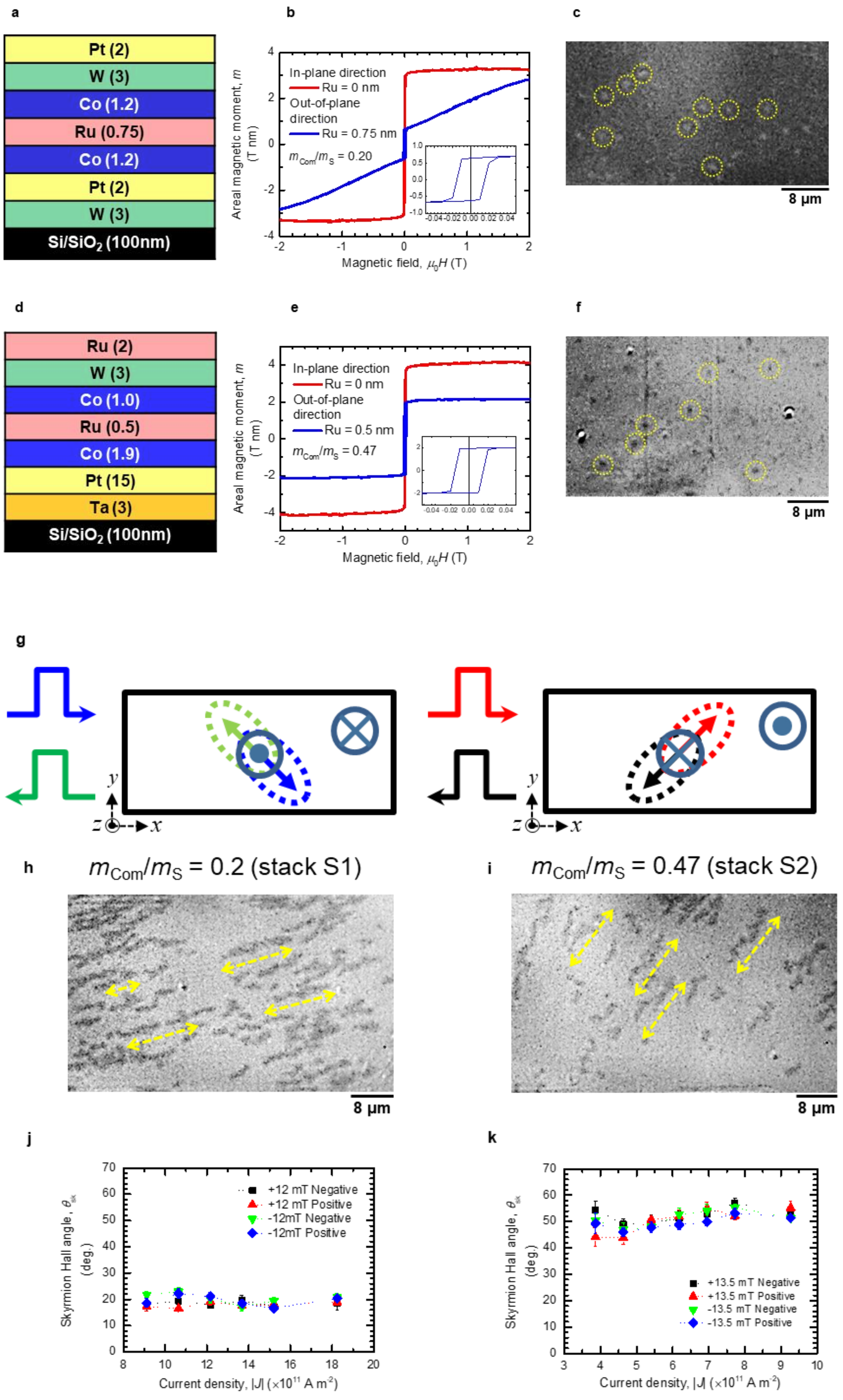

a
Pt (2)
W (3)
Co (1.2)
Ru (0.75)
Co (1.2)
Pt (2)
W (3)
Si/SiO2 (100nm)
b
In-plane direction
Ru = 0 nm
Out-of-plane direction
Ru = 0.75 nm
mCom/mS = 0.20
Areal magnetic moment, m (T nm)
Magnetic field, μ0H (T)
c
8 μm
d
Ru (2)
W (3)
Co (1.0)
Ru (0.5)
Co (1.9)
Pt (15)
Ta (3)
Si/SiO2 (100nm)
e
In-plane direction
Ru = 0 nm
Out-of-plane direction
Ru = 0.5 nm
mCom/mS = 0.47
Areal magnetic moment, m (T nm)
Magnetic field, μ0H (T)
f
8 μm
g
h
mCom/mS = 0.2 (stack S1)
8 μm
i
mCom/mS = 0.47 (stack S2)
8 μm
j
+12 mT Negative
+12 mT Positive
-12mT Negative
-12mT Positive
Skyrmion Hall angle, θsk (deg.)
Current density, |J| (×10^11 A m^-2)
k
+13.5 mT Negative
+13.5 mT Positive
-13.5 mT Negative
-13.5 mT Positive
Skyrmion Hall angle, θsk (deg.)
Current density, |J| (×10^11 A m^-2)

Supplementary Figure 6 | Current-induced elongation of synthetic antiferromagnetic skyrmion bubbles. **a**, Stack structure with Ru as interlayer exchange coupling layer for nominal FM thickness ratio = 1 (stack S1, hereafter) and **d**, for different nominal FM thickness ratio (stack S2, hereafter). **b**, *m-H* curve in stack S1 and **e**, in stack S2, where blue and red color represent *m-H* curve in perpendicular direction with optimum Ru thickness and *m-H* curve in in-plane direction without Ru, respectively. Inset shows magnified m-H_z curve around $\mu_0 H_z$ = 0 mT. **c**, Observed SyAF skyrmion bubbles in stack S1 under $\mu_0 H_z$ = –12 mT and **f**, in stack S2 under $\mu_0 H_z$ = +13.5 mT, respectively. Scale bar corresponds to 8 μm. **g**, The elongation direction of SyAF skyrmion bubble, where the direction of square wave-like arrow represents current flow direction. ⊙, ⊗ denote up and down magnetic domain, respectively. **h**, Current-induced elongation of SyAF skyrmion bubble for stack S1 under $\mu_0 H_z$ = +12 mT and **i**, for stack S2 under $\mu_0 H_z$ = –13.5 mT. Yellow broken line means elongation direction of SyAF skyrmion bubble (a guide for the eye). **j**, *J* dependence of θ_{sk} for stack S1, and **k**, for stack S2 where black square, red triangle, green inverted triangle and blue diamond means obtained results by negative *J* under positive H_z, positive *J* under positive H_z, negative *J* under negative H_z and negative *J* under positive H_z, respectively.

Supplementary Note 7
Difference in depinning field between Pt/Co/CoFeB/Ir and CoFeB/MgO systems

We investigate the depinning field in Pt/Co/CoFeB/Ir and Ta/CoFeB/MgO systems here. Starting from nucleation magnetic bubble domain, we apply pulsed perpendicular magnetic field $\mu_0 H_z$ to determine domain wall velocity υ_{DW} as is the case of Supplementary Note 2. Supplementary Figures 7a,b show typical bubble domain and H_z dependence of υ_{DW}, respectively. We clearly observe the difference in depinning field between Pt/Co/CoFeB/Ir and Ta/CoFeB/MgO systems. In general, it has been known that CoFeB systems have low depinning field due to the amorphous structure[34,35]. Although our Pt/Co/CoFeB/Ir systems contain CoFeB layer, the depinning field of those systems is still close to conventional Pt/Co structure having relatively large depinning field[35], presumably leading to the difference in skyrmion velocity for CoFeB systems[36].

In fact, the ferromagnetic skyrmion velocity in previous works[9,37,38] is one-two order different from that of our ferromagnet systems. This is attributed to the considerable difference in spin-orbit torque and depinning field as shown in Supplementary Note 3 and here, respectively. On the other hand, recent work on Pt/Co/Ir single heterostructures[39] reports the skyrmion velocity which is in good agreement with our ferromagnet Pt/Co/CoFeB/Ir single heterostructure systems. This indicates that the comparison of skyrmion velocity should be addressed carefully, and also the adequate comparison would highlight the advantage of SyAF skyrmion to ferromagnetic skyrmion for skyrmion-based application.

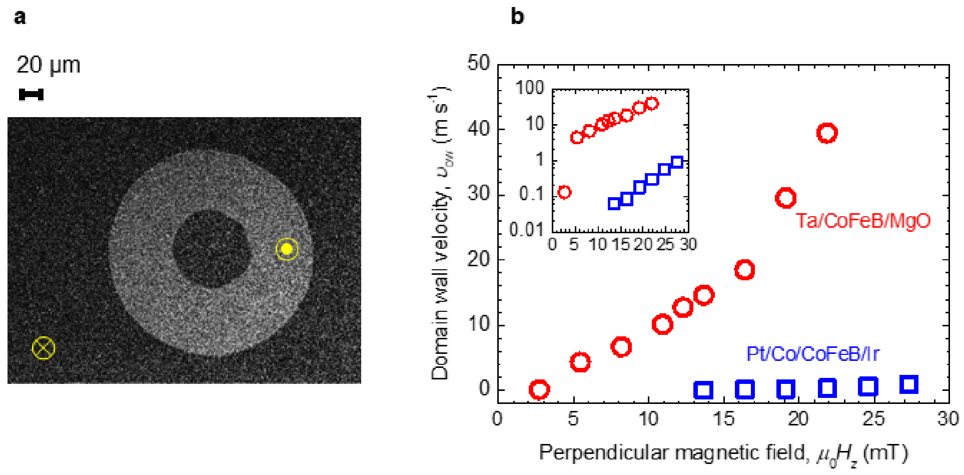

Supplementary Figure 7 | Comparison of depinning field. a. Typical MOKE image during bubble expansion measurement. **b.** Obtained velocity versus perpendicular field for Si sub./ Pt(4)/ Co(0.6)/ CoFeB (0.925)/ Ir(1.0)/ Ru(1.0) [blue square] and Si sub./ Ta(5)/ CoFeB(1.5)/ MgO(2)/ Ta(2) [Red circle]. Main panel and inset show the same result with linear and logarithmic scales, respectively.

Supplementary Note 8
Velocity limit of synthetic antiferromagnetic skyrmion bubble derived by micromagnetic simulation

In the main body, we show that achievable skyrmion velocity can be enhanced in synthetic antiferromagnetic (SyAF) systems due to the effectively compensated topological charge as well as the additive DMI and SOT. Here we show using a micromagnetic simulation that upper limit of skyrmion velocity in SyAF systems is determined by the interlayer exchange coupling. We use MuMax^3 as micromagnetic simulation software[33] as well. Supplementary Table 6 shows the list of parameters used for simulation of current-induced skyrmion motion. We do not take into account field-like torque term and Zhang-Li spin-transfer torque term, since SOT (Slonczewski-like/anti-damping like torque) is predominant factor for skyrmion bubble motion in our case as shown in the main body. Firstly, we present the exemplary current-induced motion of SyAF skyrmion with interlayer exchange coupling energy J_{int} = –0.1 mJ m^{-2} and current density along x direction J = 0.5×10^{11} A m^{-2} in Supplementary Fig. 8a. As expected, we observe the SkHE-free current-induced motion with skyrmion velocity υ_{sk} of 61 m s^{-1}. However, the SyAF-coupled skyrmion bubbles are subject to decoupling with increasing J as shown in Supplementary Fig. 8b. Skyrmion bubbles for top and bottom FM layers are compelled to undergo displacement in opposite directions owing to Magnus force (F_{y}) when exchange coupling force $F_{\mathrm{ex}} < F_{\mathrm{y}}$. Supplementary Figure 8c shows larger interlayer exchange coupling case where J_{int} equals to –2.5 mJ m^{-2} which is the largest magnitude[7] observed hitherto to our knowledge. The SyAF skyrmion bubble is not decoupled with high J = 6.0×10^{11} A m^{-2} but it experiences elongation along the direction of Magnus force. Supplementary Figure 8d summarizes υ_{sk} and aspect ratio (defined as the ratio of skyrmion size between y and x direction (y/x)) after 1 ns as a function of various J_{int}. We find that the elongation or decoupling is

inhibited with increasing J_{int} and skyrmion velocity can be reached up to about 500 m s^{-1} with aspect ratio of almost 1 for J_{int} = –2.5 mJ m^{-2}. The skyrmion velocity increases with increasing J irrespective of J_{int} and aspect ratio, where the independence of J_{int} indicates that the exchange torque[46] has no contribution due to the circular symmetry in two-dimensional space[47]. Note that the velocity decreases at high J for J_{int} = –0.1 mJ m^{-2} where skyrmion bubbles are decoupled. We presume that decoupling of skyrmion bubbles determines the upper limit of skyrmion velocities which can be utilized in skyrmion based memory devices.

Supplementary Table 6 | Parameter list for current-induced motion of synthetic antiferromagnetic skyrmion bubble in micromagnetic simulation

World size (top, bottom thickness)	512x512x2 nm^3 (1 nm, 1 nm)
Cell size	1x1x1 nm^3
Periodic boundary condition	8x8x0
Spontaneous magnetization, M_S	1.2 T
Uniaxial anisotropy energy density, K_U	0.85 MJ m^{-3}
Interfacial DMI, D_I	-2.0 mJ m^{-2}
Exchange stiffness, A_S	10 pJ m^{-1}
Gilbert damping, α	0.1
Spin Hall angle θ_{SH}	0.25

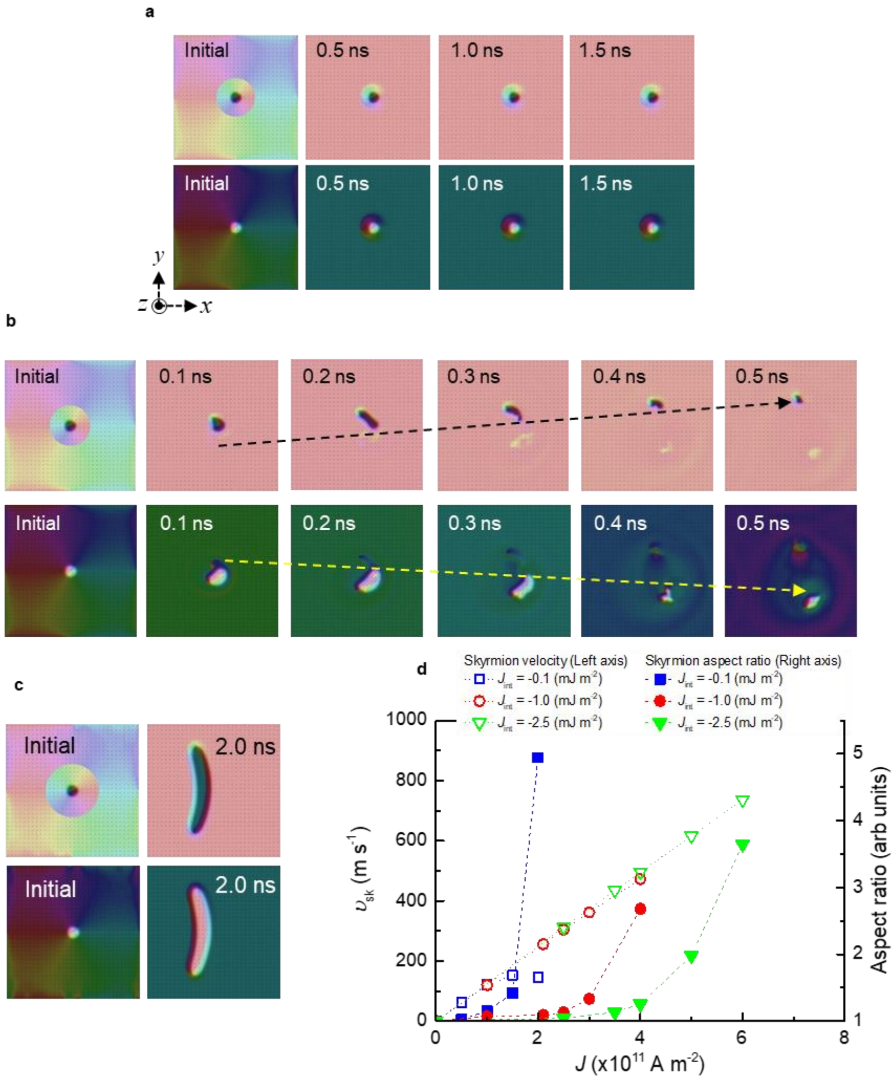

Supplementary Figure 8 | Micromagnetic simulation for current-induced motion of synthetic antiferromagnetic skyrmion bubble. a, Current-induced motion of SyAF skyrmion bubble with J_{int} = –0.1 mJ m^{-2} and spin-current density J = 0.5×10^{11} A m^{-2} captured per 0.5 ns, where top and bottom panels represent top and bottom ferromagnetic layer, respectively. **b**, Current-induced motion of SyAF skyrmion bubble with J_{int} = –0.1 mJ m^{-2} and J = 2.0×10^{11} A m^{-2}. Subsequent images were captured in 0.1 ns time-step during simulations. **c**, Current-induced motion of SyAF skyrmion bubble with J_{int} = –2.5 mJ m^{-2} and J = 6.0×10^{11} A m^{-2} for initial state (left panels) and after 2.0 ns (right panels). **d**, J dependence of υ_{sk} (open symbol, left axis) and aspect ratio (filled symbol, right axis) as a function of various J_{int}, where blue, red and yellow green denote –0.1, –1.0 and –2.5 mJ m^{-2}, respectively.

Supplementary References

1. Grünberg, P., Schreiber, R., Pang, Y., Brodsky, M. B. & Sowers, H. Layered Magnetic Structures: Evidence for Antiferromagnetic Coupling of Fe Layers across Cr Interlayers. *Physical Review Letters* **57**, 2442–2445 (1986).
2. Slonczewski, J. C. Conductance and exchange coupling of two ferromagnets separated by a tunneling barrier. *Physical Review B* **39**, 6995–7002 (1989).
3. Parkin, S. S. P., More, N. & Roche, K. P. Oscillations in exchange coupling and magnetoresistance in metallic superlattice structures: Co/Ru, Co/Cr, and Fe/Cr. *Physical Review Letters* **64**, 2304–2307 (1990).
4. Bruno, P. Theory of interlayer magnetic coupling. *Physical Review B* **52**, 411–439 (1995).
5. Duine, R. A., Lee, K.-J., Parkin, S. S. P. & Stiles, M. D. Synthetic antiferromagnetic spintronics. *Nature Physics* **14**, 217–219 (2018).
6. Hayakawa, J. *et al.* Current-Induced Magnetization Switching in MgO Barrier Based Magnetic Tunnel Junctions with CoFeB/Ru/CoFeB Synthetic Ferrimagnetic Free Layer. *Japanese Journal of Applied Physics* **45**, L1057–L1060 (2006).
7. Yakushiji, K., Sugihara, A., Fukushima, A., Kubota, H. & Yuasa, S. Very strong antiferromagnetic interlayer exchange coupling with iridium spacer layer for perpendicular magnetic tunnel junctions. *Applied Physics Letters* **110**, 092406 (2017).
8. Itoh, H., Yanagihara, H., Suzuki, K. & Kita, E. Coexistence of the uniaxial anisotropy and the antiferromagnetic coupling in Co/Ir(111) superlattices. *Journal of Magnetism and Magnetic Materials* **257**, 184–189 (2003).
9. Woo, S. *et al.* Observation of room-temperature magnetic skyrmions and their current-driven dynamics in ultrathin metallic ferromagnets. *Nature Materials* **15**, 501–506 (2016).
10. Soumyanarayanan, A. *et al.* Tunable room-temperature magnetic skyrmions in Ir/Fe/Co/Pt multilayers. *Nature Materials* **16**, 898–904 (2017).

11. Maccariello, D. *et al.* Electrical detection of single magnetic skyrmions in metallic multilayers at room temperature. *Nature Nanotech* **13**, 233–237 (2018).
12. Je, S.-G. *et al.* Asymmetric magnetic domain-wall motion by the Dzyaloshinskii-Moriya interaction. *Physical Review B* **88**, 214401 (2013).
13. Lavrijsen, R. *et al.* Asymmetric magnetic bubble expansion under in-plane field in Pt/Co/Pt: Effect of interface engineering. *Physical Review B* **91**, 104414 (2015).
14. Emori, S., Bauer, U., Ahn, S.-M., Martinez, E. & Beach, G. S. D. Current-driven dynamics of chiral ferromagnetic domain walls. *Nature Materials* **12**, 611–616 (2013).
15. Yang, H., Thiaville, A., Rohart, S., Fert, A. & Chshiev, M. Anatomy of Dzyaloshinskii-Moriya Interaction at Co/Pt Interfaces. *Physical Review Letters* **115**, 267210 (2015).
16. Ma, X. *et al.* Interfacial Dzyaloshinskii-Moriya Interaction: Effect of 5d Band Filling and Correlation with Spin Mixing Conductance. *Physical Review Letters* **120**, 157204 (2018).
17. Kim, S. *et al.* Correlation of the Dzyaloshinskii–Moriya interaction with Heisenberg exchange and orbital asphericity. *Nature Communications* **9**, 1648 (2018).
18. Jué, E. *et al.* Chiral damping of magnetic domain walls. *Nature Materials* **15**, 272–277 (2016).
19. Kim, D.-Y. *et al.* Chirality-induced antisymmetry in magnetic domain wall speed. *NPG Asia Materials* **10**, e464 (2018).
20. Shahbazi, K. *et al.* Domain-wall motion and interfacial Dzyaloshinskii-Moriya interactions in Pt/Co/Ir(tIr)/Ta multilayers. *Physical Review B* **99**, 094409 (2019).
21. Vaňatka, M. *et al.* Velocity asymmetry of Dzyaloshinskii domain walls in the creep and flow regimes. *J. Phys.: Condens. Matter* **27**, 326002 (2015).
22. Dohi, T., DuttaGupta, S., Fukami, S. & Ohno, H. Reversal of domain wall chirality with ferromagnet thickness in W/(Co)FeB/MgO systems. *Applied Physics Letters* **114**, 042405 (2019).

23. Kim, D.-H., Kim, D.-Y., Yoo, S.-C., Min, B.-C. & Choe, S.-B. Universality of Dzyaloshinskii-Moriya interaction effect over domain-wall creep and flow regimes. *Physical Review B* **99**, 134401 (2019).
24. Pai, C.-F., Mann, M., Tan, A. J. & Beach, G. S. D. Determination of spin torque efficiencies in heterostructures with perpendicular magnetic anisotropy. *Phys. Rev. B* **93**, 144409 (2016).
25. Ishikuro, Y., Kawaguchi, M., Kato, N., Lau, Y.-C. & Hayashi, M. Dzyaloshinskii-Moriya interaction and spin-orbit torque at the Ir/Co interface. *Phys. Rev. B* **99**, 134421 (2019).
26. Jamali, M. *et al.* Spin-Orbit Torques in Co/Pd Multilayer Nanowires. *Phys. Rev. Lett.* **111**, 246602 (2013).
27. Huang, K.-F., Wang, D.-S., Lin, H.-H. & Lai, C.-H. Engineering spin-orbit torque in Co/Pt multilayers with perpendicular magnetic anisotropy. *Appl. Phys. Lett.* **107**, 232407 (2015).
28. Thiaville, A., Rohart, S., Jué, É., Cros, V. & Fert, A. Dynamics of Dzyaloshinskii domain walls in ultrathin magnetic films. *EPL (Europhysics Letters)* **100**, 57002 (2012).
29. Ryu, K.-S., Thomas, L., Yang, S.-H. & Parkin, S. Chiral spin torque at magnetic domain walls. *Nature Nanotechnology* **8**, 527–533 (2013).
30. Dohi, T., Kanai, S., Okada, A., Matsukura, F. & Ohno, H. Effect of electric-field modulation of magnetic parameters on domain structure in MgO/CoFeB. *AIP Advances* **6**, 075017 (2016).
31. Dohi, T., Kanai, S., Matsukura, F. & Ohno, H. Electric-field effect on spin-wave resonance in a nanoscale CoFeB/MgO magnetic tunnel junction. *Appl. Phys. Lett.* **111**, 072403 (2017).
32. Ichikawa, N. *et al.* Non-linear variation of domain period under electric field in demagnetized CoFeB/MgO stacks with perpendicular easy axis. *Appl. Phys. Lett.* **112**, 202402 (2018).
33. Vansteenkiste, A. *et al.* The design and verification of MuMax3. *AIP Advances* **4**, 107133 (2014).
34. Burrowes, C. *et al.* Low depinning fields in Ta-CoFeB-MgO ultrathin films with perpendicular magnetic anisotropy. *Appl. Phys. Lett.* **103**, 182401 (2013).

35. Jeudy, V., Díaz Pardo, R., Savero Torres, W., Bustingorry, S. & Kolton, A. B. Pinning of domain walls in thin ferromagnetic films. *Physical Review B* **98**, (2018).

36. Legrand, W. *et al.* Room-Temperature Current-Induced Generation and Motion of sub-100 nm Skyrmions. *Nano Lett.* **17**, 2703–2712 (2017).

37. Jiang, W. *et al.* Direct observation of the skyrmion Hall effect. *Nature Physics* **13**, 162–169 (2017).

38. Litzius, K. *et al.* Skyrmion Hall effect revealed by direct time-resolved X-ray microscopy. *Nature Physics* **13**, 170–175 (2017).

39. Sugimoto, S., Kasai, S., Anokhin, E., Takahashi, Y. & Tokura, Y. Nonequilibrium skyrmion accumulation induced by direct current in Ir/Co/Pt heterostructure. *Appl. Phys. Express* **12**, 073002 (2019).

40. Hirata, Y. *et al.* Vanishing skyrmion Hall effect at the angular momentum compensation temperature of a ferrimagnet. *Nature Nanotechnology* **14**, 232–236 (2019).

41. Jiang, W. *et al.* Blowing magnetic skyrmion bubbles. *Science* **349**, 283–286 (2015).

42. Büttner, F. *et al.* Field-free deterministic ultrafast creation of magnetic skyrmions by spin–orbit torques. *Nature Nanotechnology* **12**, 1040–1044 (2017).

43. Romming, N. *et al.* Writing and Deleting Single Magnetic Skyrmions. *Science* **341**, 636–639 (2013).

44. Sampaio, J., Cros, V., Rohart, S., Thiaville, A. & Fert, A. Nucleation, stability and current-induced motion of isolated magnetic skyrmions in nanostructures. *Nature Nanotechnology* **8**, 839–844 (2013).

45. Tserkovnyak, Y., Brataas, A., Bauer, G. E. W. & Halperin, B. I. Nonlocal magnetization dynamics in ferromagnetic heterostructures. *Rev. Mod. Phys.* **77**, 1375–1421 (2005).

46. Yang, S.-H., Ryu, K.-S. & Parkin, S. Domain-wall velocities of up to 750 m s^{-1} driven by exchange-coupling torque in synthetic antiferromagnets. *Nature Nanotechnology* **10**, 221–226 (2015).

47. Büttner, F., Lemesh, I. & Beach, G. S. D. Theory of isolated magnetic skyrmions: From fundamentals to room temperature applications. *Scientific Reports* **8**, 4464 (2018).
